# Supplementary material for: Accelerated body size evolution in upland environments is correlated with recent speciation in South American freshwater fishes
Source: Nat Commun. 2023 Sep 28;14:6070. doi: 10.1038/s41467-023-41812-7 (PMC10539357; doi:10.1038/s41467-023-41812-7)
Supplement: Supplementary file 1 — Supplementary Information [file 41467_2023_41812_MOESM1_ESM.pdf]

Supplementary Materials for

Accelerated body size evolution in upland environments is correlated with recent speciation in South American freshwater fishes

Felipe O. Cerezer[1,2,*], Cristian S. Dambros[2], Marco T. P. Coelho[1], Fernanda A. S. Cassemiro[3], Elisa Barreto[1], James S. Albert[4], Rafael O. Wüest[1], & Catherine H. Graham[1]

[1]Swiss Federal Research Institute for Forest, Snow, and Landscape (WSL), Birmensdorf, Switzerland; [2]Programa de Pós-Graduação em Biodiversidade Animal, Departamento de Ecologia e Evolução, Universidade Federal de Santa Maria, Santa Maria, Brazil; [3]Programa de Pós-Graduação em Ecologia e Evolução, Universidade Federal de Goiás, Goiânia, Brazil; [4]Department of Biology, University of Louisiana at Lafayette, Lafayette, USA

[*]Corresponding author: cerezerfelipe@gmail.com

This PDF file includes:

Supplementary Note 1: Sensitivity Analyses ….……………………………………………….2
Supplementary Note 2: Extended Methods ………………………………………………….9
Supplementary Figures 1 to 41 …………………………………………………………..….15
Supplementary Tables 1 to 3 ……………………………….…………………………...56
Supplementary References ...……………………………………………………………..59

Supplementary Note 1: Sensitivity Analyses

Speciation rate estimates, alternative methods, and phylogenetic uncertainty. The quantification of speciation rates has been the subject of considerable debate[1], and to alleviate these issues, we evaluated the robustness of our speciation rate estimates across different sensitivity scenarios. In this study, we focused on estimates near the present, known as tip-based rates, as it has been shown that estimates from deep time are less reliable[2,3].

To ensure that our estimates were well-supported by the data, we used three different methods with distinct underlying assumptions (BAMM, DR statistic, and MiSSE). The fact that our tip speciation rate estimates were highly correlated across all three methods (Figs. S4-S5) suggests that they are reliable and grounded in the data. Given the robustness of our results, we chose to focus on the estimates obtained from the BAMM method[4], which has been demonstrated to outperform other methods and is widely regarded as a highly reliable and accurate approach[5,6].

We assessed geographic patterns of speciation rates across South American sub-basins by averaging tip rates based on species list occurrences for each sub-basin. While the common practice is to use community-weighted mean, we also explored alternative summary statistics due to the potential limitations of using a mean value to represent a variable with high variability. Therefore, we calculated the median, minimum, and maximum values of speciation rates across sub-basins to gain a more comprehensive understanding of the distribution of speciation rates across regions. Our analysis revealed spatial patterns that were consistent with our main findings, demonstrating regions with both low and high speciation rates (Fig. S20), and supporting the relative importance of different predictors in driving speciation (Fig. S21).

In order to assess the reliability and robustness of BAMM tip estimates, we also compared our BAMM tip estimates to those obtained from different phylogenetic hypotheses. Specifically, we ran BAMM using alternative phylogenies and compared their tip rates to ours. We first compared our estimates from the taxonomic-genetic tree with the same phylogeny, but only considering species with genetic data[7]. We also performed this comparison with the largest ray-finned fish supertree[8], considering 1) only species with genetic data, and 2) with only South American species. Additionally, we compared our estimates to those obtained from smaller, clade-specific trees: 1) poecillidae[9], 2) cichlid[10], and 3) characoid[11] fishes. Even after conducting a thorough comparison of our estimates with those derived from different phylogenies, we observed significant correlations ranging from moderate to high (Fig. S7). This finding highlights the robustness and reliability of our estimates, indicating a consistent pattern across diverse evolutionary frameworks. However, it is important to acknowledge that the estimates of evolutionary rates also exhibit variance, which is worth mentioning. Deviations from our tip rate estimates could arise due to at least two underlying factors: 1) variations stemming from the chosen methodologies for phylogeny construction and taxon sampling[12], and 2) the presence of several deterministic events that cannot be directly measured[13]. Nevertheless, our estimates were derived from the most current and comprehensive reconstruction of phylogenetic relationships among South American fish species[7].

Speciation rates through time. Our study aimed to investigate the relationship between a set of biotic and abiotic factors and speciation rates over geography. However, we also made some temporal observations related to evolutionary rates that are worth mentioning. We used rates-through-time plots to observe that speciation rates remained steady until the late Cretaceous when they substantially started to increase (Fig. S1). Interestingly, we also found some consistencies when parallelizing speciation rate events with the temporal

variation of morphological evolution rates (Fig. S3). More importantly, rates of body size evolution were the only trait that closely followed a similar increase to speciation rates (Fig. S1). This was notably observed from the Paleogene when body size evolution accelerated while the remaining traits were observed to decelerate during this time (Fig. S3). Taken together, these temporal observations could help explain the positive correlation between speciation rates and body size evolution or the negative correlation between speciation and oral gape position and relative maxillary length evolution that we observed in our spatial approach (Fig. 2).

It is also worth noting that we were unable to apply a temporal approach in a manner similar to our spatial approach due to the lack of data. For example, reliable climate data covering our phylogeny's time frame is scarce (~250 mya), as is data on habitat conditions (e.g. area, elevation, soil diversity, surface runoff) and species richness. This makes a potential temporal approach impractical and not comparable to our spatial approach since we would be missing key variables. Therefore, we focused on the spatial approach and discuss our findings within this context. Although there have been attempts to address these temporal questions[7,14], future studies that incorporate a temporal approach with more comprehensive data may provide further insights into the evolutionary history of the studied taxa.

Morphological evolution estimates, alternative methods, and incomplete taxonomic sampling. To assess the robustness of our estimates of morphological evolution, we used different metrics and checked for the effects of incomplete taxonomic coverage. Specifically, we quantified species-specific rates of five morphological traits using two robust metrics: the BAMM trait module and BayesTraits. We found a strong correlation between tip-based rates estimated using these two metrics for all five traits (Figs. S8-S12), suggesting that the recovered estimates are unlikely to be biased towards a specific

method. However, we chose to use BAMM method for trait evolution due to its ability to model multiple time-dependent, gradual rate changes, providing a representation of continuous rate-variation processes with occasional jumps[4]. The results from BayesTraits models were considered supplementary to our analysis.

Recent studies have indicated that incomplete taxonomic coverage could hinder the ability to accurately estimate evolutionary rates[12]. Therefore, we tested the robustness of our morphological rate estimates to variations in taxonomic sampling, given that the proportion of sampled species in our study was incomplete and variable (Table S2). To address this, we extracted data on maximum body length for 4,228 species (85% of the South American ichthyofauna) from Fishbase[15] and used BAMM to re-estimate speciation rates and body size evolution. Surprisingly, we found that the strong correlation between speciation rates and body size evolution still persisted in this massive dataset (Fig. S13). Thus, we are confident in asserting that our estimates are reliable and not greatly affected by taxonomic sampling effects. It is essential to note that we could not use a higher number of species than those obtained from Fishbase because the data for the other four traits were unavailable in this dataset.

Species-area relationships, the effects of low species number, and wide-ranging species. We investigated the potential influence of the species-area relationship[16], which is described by the power function SD = SR/Az, where SD is the species density (i.e., the number of species per unit area), SR is the total number of species in area A, and z is the species-area scaling exponent (i.e., the slope of the species-area regression). We tested the impact of poor-species regions on our conclusions by excluding sub-basins with a small number of species (i.e. 10, 15, and 20 species). Our analysis revealed that species density predicted speciation rates similarly when using raw species richness or accounting for the species-area relationship (Fig. S33-S34).

Finally, we evaluated the potential impact of wide-ranging species when averaging speciation rates across space. To address this issue, we weighted tip rates by species range size when averaging speciation rates[17]. We calculated species range size by summing the geographic areas of all sub-basins where the species occurs. We observed that the resulting spatial patterns of speciation rates were highly congruent with those reported in the main text (Fig. S36). Furthermore, we found that the same predictors remained important even after removing the excessive influence of species with wide distribution (Fig. S36). Overall, we are confident in asserting that our conclusions were not affected by sub-basins with low species richness or species with wide range size.

Paleotemperature and temperature-dependent speciation model. To test the impact of historical temperatures on speciation rates, we adopted two approaches. First, we gathered paleotemperature data from the CHELSA database[18] for the Pliocene (ca. 3.3 Mya) and the Last Glacial Maximum (LGM; ca. 21 ka) epochs, at a 2.5 arc-minute resolution. Second, we implemented a temperature-dependent speciation model using the RPANDA R package[19]. For this model, we used temperature fluctuations during the entire Cenozoic era[20] (~66 mya) and specifically focused on freshwater clades more diverse than 50 species that arose during this time frame. We divided this approach into clades because reliable paleotemperature data were not available for our phylogenetic time coverage (~ 250 mya). Our results suggest that paleotemperature has left imprints on speciation rates, consistent with findings showing that current temperatures also have a positive effect on speciation rates. Specifically, we found that both the Miocene and LGM historical temperatures were positively associated with speciation rates (Fig. S14). Our model-based phylogenetic comparative method supported these results, showing that 72% of the selected clades (8 out of 11 clades) exhibit a positive effect of historical temperature on speciation rates (Fig. S15).

Assessing the effects of multiple habitat-related metrics on speciation rates. There are various ways to represent elevation and topographic complexity. To address this variation, we collected different proxies related to topography. We obtained mean elevation, standard deviation in elevation, terrain slope, and basin relief for each sub-basin using the HydroATLAS[21] database at a 15-arc-second resolution. Our analysis showed that these different proxies similarly predicted speciation rates and mean elevation was the best predictor of speciation rates among them (Fig. S38).

Spatial autocorrelation. Observations in ecology are often related to each other across space, and this phenomenon can cause spurious relationships in statistical analyses[22]. To test for this spatial autocorrelation, we calculated Moran's I[23] based on the residual speciation rates (i.e., the differences between the actual speciation rates and the values predicted by a multiple linear regression using the eleven predictors). Our analysis revealed that the residuals from the non-spatial multiple regression exhibited a low spatial autocorrelation pattern (Global Moran's I = -0.003, P = 0.423; see Figure S41) and are unlikely to affect the validity of our findings.

Assessing the impact of biological outliers on phylogenetic and geographic estimates. Outliers can have a significant influence on regression estimates[24]. In our study, we observed that our speciation estimates, both in phylogenetic and spatial perspectives, were influenced by outliers represented by the *Orestias* species. The genus *Orestias* has been known to be a speciose group, sometimes comparable to cichlid species, due to incipient speciose processes[25,26]. To address this issue, we employed three different approaches. First, we removed *Orestias* species from the phylogeny and re-ran the BAMM analysis. Second, we excluded *Orestias* species when calculating the mean

speciation rates across sub-basins, which could affect the mean distribution in their presence. Third, we removed the five sub-basins that were predominantly occupied by *Orestias* species and exhibited remarkable speciation rates. Notably, regardless of the approach used to exclude this small clade, we found that the removal of the exceptionally-speciating *Orestias* species did not impede the ability to identify high speciation rates in other clades (Fig. S16). Moreover, the exclusion of *Orestias* species did not change the speciation rates distribution across sub-basins and, consequently, did not under- or overestimate the main determinants of speciation rates (Fig. S17-19).

Supplementary Note 2: Extended Methods

Geographic occurrences and phylogenetic data. Our study utilized an extensive collection of data on South American freshwater fish[7], encompassing the presence or absence of 4,967 species across 460 sub-basins, as delimited by the HydroBASINS framework at level 5[27]. This dataset was meticulously compiled from diverse sources, including web repositories such as GBIF and specieslink, as well as literature sources. To ensure data accuracy and reliability, rigorous procedures were implemented, including the removal of georeferencing errors, exotic species and migratory species. Furthermore, Cassemiro et al.[7] accounted for heterogeneities in sampling efforts across species distributions using simulation approaches (i.e. completeness index for each drainage basin). This index estimated the probability of sampling a new species not previously observed in the focal basin, thereby addressing potential sampling effort variations across different areas. As a result, this dataset maintained high data quality standards and minimized potential biases in species distributions.

To quantify evolutionary rates, we used a newly compiled, time-calibrated tree of Neotropical freshwater fishes[7]. This comprehensive tree was based on 5,984 terminal taxa, including 3,169 species with available genetic data from 51 independently aligned and trimmed markers. Additionally, the tree incorporated 31 fossil-constrained nodes and 2,815 species inserted by taxonomic imputation, making it a state-of-the-art summary of the current understanding of phylogenetic relationships among Neotropical freshwater fishes. Finally, we carefully selected species by aligning their geographic occurrence and phylogeny with available morphological data (as shown below), resulting in a total of 2,638 species that were used in our study.

Estimating speciation rates and assessing their reliability. Our investigation of diversification patterns across different fish lineages involved using three alternative statistical methods. We employed Bayesian Analysis of Macroevolutionary Mixtures (BAMM)[4], diversification rate (DR)[28] statistics, and Missing State Speciation and Extinction (MiSSE) metric[2] to estimate species-specific diversification rates (tip-based estimates). BAMM is a widely used tool in evolutionary biology for studying species diversification over time, allowing for the identification of shifts in diversification rates, estimation of speciation and extinction rates, and exploration of trait-diversification relationships[4,5]. The DR statistic is calculated as the inverse of equal split rates, providing insights into the relative rates of speciation and how they may have changed across lineages or over time[5,28]. MiSSE represents a recently developed method that builds upon a trait-free version of the HiSSE framework[2]. It allows researchers to conduct comprehensive investigations and analyses of diversification rates at the tips of the phylogeny, by considering a wide range of scenarios of speciation and extinction

To obtain BAMM estimates, we conducted extensive simulations using Markov Chain Monte Carlo (MCMC) with four independent chains and a total of 20 million generations, with sampling every 2,000 generations. We also accounted for incomplete taxon sampling by using a global sampling fraction of 0.53[29]. Prior settings for speciation and extinction were obtained using the ‘setBAMMpriors’ function in the R *BAMMtools* package[30], with values of: expectedNumberOfShifts = 1.0, lambdaInitPrior and muInitPrior = 7.0435, and lambdaShiftPrior = 0.0045. To ensure reliable results, we discarded the first 25% of MCMC samples as burn-in and confirmed the effective sample size of all parameters was above 200 using the R *CODA* package[31]. The DR statistic was calculated as the inverse of evolutionary distinctiveness using the ‘evol.distinct’ function from the R *picante* package[32]. Furthermore, the MiSSE model was applied using the 'MiSSEGreedy' and 'generateMiSSEGreedyCombinations' functions from the R *hisse* package[33]. Our

analysis employed specific parameters, including ‘stopdeltaAICc = 10’, ‘chunk.size = 10’, and a sampling fraction of ‘0.53’. To obtain an overview of reconstructions from a collection of models, we utilized the 'GetModelAveRates' function to derive tip speciation rates.

For both BAMM, DR, and MiSSE estimators, we extracted tip-based speciation rates, which are considered more robust for recent time estimates compared to deep-time estimates[2,3]. While both speciation and extinction processes were estimated through the phylogeny, our reporting mainly focused on speciation estimates due to challenges in estimating extinction rates from extant phylogenies[34,35]. Consequently, we explored spatial patterns from the three methods by averaging tip speciation rate estimates among co-occurring species in each subdrainage basin. We averaged speciation rates using the *rgdal*[36] and *SYNCSA*[37] packages.

Morphological traits, their rates of evolution, and robustness. To capture the ecological, physiological, and behavioral dimensions of fish species, we carefully selected five morphological traits from the Fishmorph database[38]. These quantitative traits are: body elongation (BEL), relative eye size (RES), oral gape position (OGP), relative maxillary length (RML), and maximum body length (MBL). The calculations for these traits are as follows[38]: 1) BEL = standard length divided by the maximum body length; 2) RES = vertical diameter of the eye divided by head depth at the vertical of the eye; 3) OGP = vertical distance from the top of the mouth to the bottom of the body divided by maximum body length; 4) RML = length from snout to the corner of the mouth divided by head depth at the vertical of the eye; and 5) MBL = maximum adult length in centimeters. Each of these traits has been linked to crucial aspects of fish biology[39–43], such as swimming performance, visual acuity, feeding behavior, and metabolic rate. To ensure statistical rigor, we log10-transformed body size estimates prior to estimating evolutionary rates, following recommendations for size data[44].

To conduct our analyses, we pruned the phylogeny of Neotropical freshwater fishes to match the South American species with available morphological data, resulting in a dataset of 2,638 species (53% of the South American species). Due to missing trait data for some species (Figs. S22-S26), we used the *Rphylopars* package to impute these values leveraging phylogenetic information as imputation criteria[45]. This method has been recommended as it outperforms other approaches[46]. We further demonstrated that species lacking any of the five morphological data were not phylogenetically clustered, indicating that our results are not biased towards or against any particular fish clade.

To estimate the phenotypic evolutionary rate for each of the five morphological traits, we utilized two reliable methods: the BAMM 'trait' module[4] and BayesTraits[47]. These methods have been demonstrated to be highly accurate for per-lineage rate estimates[6]. By using both approaches, we increased the robustness of our estimates and obtained more confidence in the accuracy of our results. For the BAMM trait analysis, we ran four independent Markov Chain Monte Carlo (MCMC) chains, each consisting of 100 million generations, with sampling conducted every 10,000 generations, and discarded the first 25% of samples as burn-in. These long chains were performed to ensure reliable estimates, confirmed by the effective sample size of all parameters exceeding 200 (the outputs can be verified using the provided codes and data). We used the BayesTraits analysis to detect changes in the rate of evolution of continuous traits[47]. Specifically, we utilized version 2.02 of the variable rates model and employed a MCMC sampler run with 3,000,000 iterations, sampling every 1,000 iterations, and discarded the first 600,000 iterations as a burn-in period. This was sufficient to ensure model convergence. The variable rates model detects shifts in the rate of evolution by rescaling branch lengths, where trait change differs from what is expected under a uniform Brownian motion model. The calculated scalars indicate the amount of acceleration or deceleration relative to the background rate on the branch of interest[48]. To obtain the final parameter results, we used

the variable rates post-processor[48], which derives branch-specific values based on the mean scalar parameter.

Our findings consistently demonstrated convergent tip-based estimates of morphological evolution when using both the BAMM trait and BayesTraits methods (see Extended Sensitivity analysis section below). Based on this robust evidence, we focused our subsequent analyses on the rates of morphological evolution derived from BAMM, considering the results from BayesTraits as supplementary. In a similar manner to how we calculated speciation rates, we utilized tip-based evolutionary rates obtained from BAMM to determine the mean rates of morphological evolution for each sub-basin, based on their respective species assemblages (Figs. S27-S31).

Species diversity. In order to assess species diversity in each subdrainage basin, we calculated the total number of fish species co-occurring in each basin (Fig. S32).

Climate variables. Climatic conditions are believed to influence speciation by potentially impacting metabolic rates[49] and/or the size of species pools[50]. To comprehensively assess the relationship between climate and speciation rates, we obtained data on four key variables directly related to climate: (i) annual mean temperature, (ii) annual mean precipitation, (iii) actual evapotranspiration, and (iv) land surface runoff. Data on annual mean temperature and precipitation were acquired from the WorldClim Version 1 database at a resolution of 2.5 arc-minutes, representing the mean from 1970-2000. The air temperature was used as a proxy for water temperature, as large-scale water temperature data were unavailable. Data on actual evapotranspiration and land surface runoff were extracted from the HydroATLAS database at a resolution of 15 arc-seconds[21]. The mean values of the climate variables were calculated for each of the 460 sub-basins (Fig. S37)

using the 'extract' function from the *raster* package[51]. An overview of the climate variables and data treatment can be found in Table 3.

Habitat variables. In order to accurately represent the habitat characteristics of the sub-basins, we considered four key variables, including: (i) mean elevation, (ii) geographic area of the sub-basin measured in square meters, (iii) stream gradient, and (iv) soil diversity. These variables are recognized as significant drivers of speciation rates, either for capturing niche dimensions of fish species[52–54] or for representing hydro-physiographic features that are primarily associated with isolation and environmental heterogeneity[55,56]. Data on elevation were obtained from the WorldClim Version 1 database at a resolution of 2.5 arc-minutes. Geographic area of the sub-basin was acquired from HydroBASINS at level 5[27], while stream gradient and soil types data were extracted from HydroATLAS at a resolution of 15 arc-seconds[21]. Soil types data included: clay fraction, silt fraction, sand fraction, organic carbon content, soil water content, lithological classes, karst area extent, and soil erosion. To quantify soil diversity, we utilized the Shannon diversity index based on substrate types and soil conditions within each sub-basin. The Shannon index was computed using the *vegan* package[57] in R. The mean values of the habitat variables were calculated for each of the 460 sub-basins (Fig. S39) using the 'extract' function from the *raster* package[51]. An overview of the climate variables and data treatment can be found in Table 3.

Statistical analysis. To examine the relationships among recent speciation rates and various biotic and abiotic factors, including rates of trait evolution, species diversity, climate, and habitat variables, we conducted a multiple linear regression analysis. Prior to the regression analysis, we assessed multicollinearity among predictors using the variance inflation factor (VIF) from the *car* package[58] in R. We identified three variables with high

multicollinearity (rate of evolution of relative eye size, annual precipitation, and actual evapotranspiration) (Fig. S40), which were subsequently removed from the main analyses. This left us with eleven variables that had low multicollinearity (VIF < 5; see Table S1). To ensure comparability of effect sizes among predictors, we standardized both response and predictor variables using z-score standardization (mean = 0; sd = 1). This procedure was performed using the “scale” R base function. Sub-basin area was log-transformed prior to z-score standardization. To gain a more detailed understanding of how each predictor variable contributes to the total variance in speciation rates, we utilized hierarchical partitioning with the metric lmg method. This method was implemented using the R package *relaimpo*[59]. Hierarchical partitioning is a statistical approach that decomposes the explained variance of a model into non-negative contributions from each predictor variable[60]. This allows us to evaluate the relative importance of each variable in the linear models and elucidate their individual contributions to the overall variance in speciation rates.

Additionally, we used variance partitioning to investigate the unique and shared effects of four major mechanisms as predictors of speciation rates. By isolating the effects of each mechanism and examining their individual contributions to the variance in speciation rates, we were able to identify which major mechanisms had the strongest impact on speciation rates and whether they acted independently or in combination with other mechanisms. More specifically, the variables were grouped into four categories: (1) rates of morphological evolution (rates of body elongation, maximum body length, oral gape position, and relative maxillary length evolution), (2) diversity-dependent speciation (species diversity), (3) climate-driven speciation (temperature and surface runoff), and (4) habitat-driven speciation (elevation, stream gradient, soil diversity, and area). Variance partitioning was performed through the ‘varpart’ function of the *vegan* package[57].

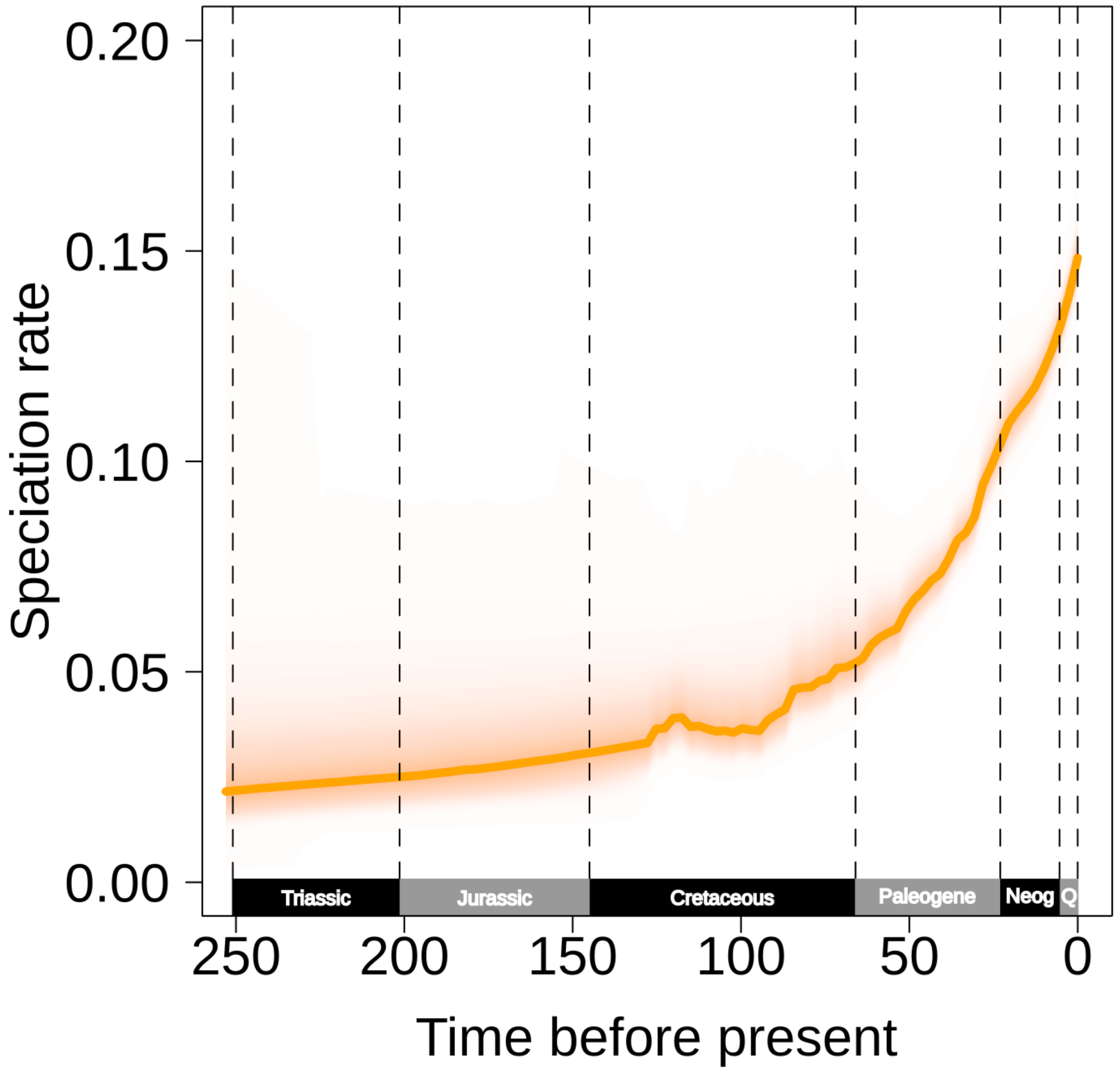

Figure S1. Temporal dynamics of fish speciation in South America, based on BAMM analysis. The graph displays the speciation-through-time pattern with corresponding confident intervals. The Neogene and Quaternary periods are abbreviated as 'Neog' and 'Q', respectively.

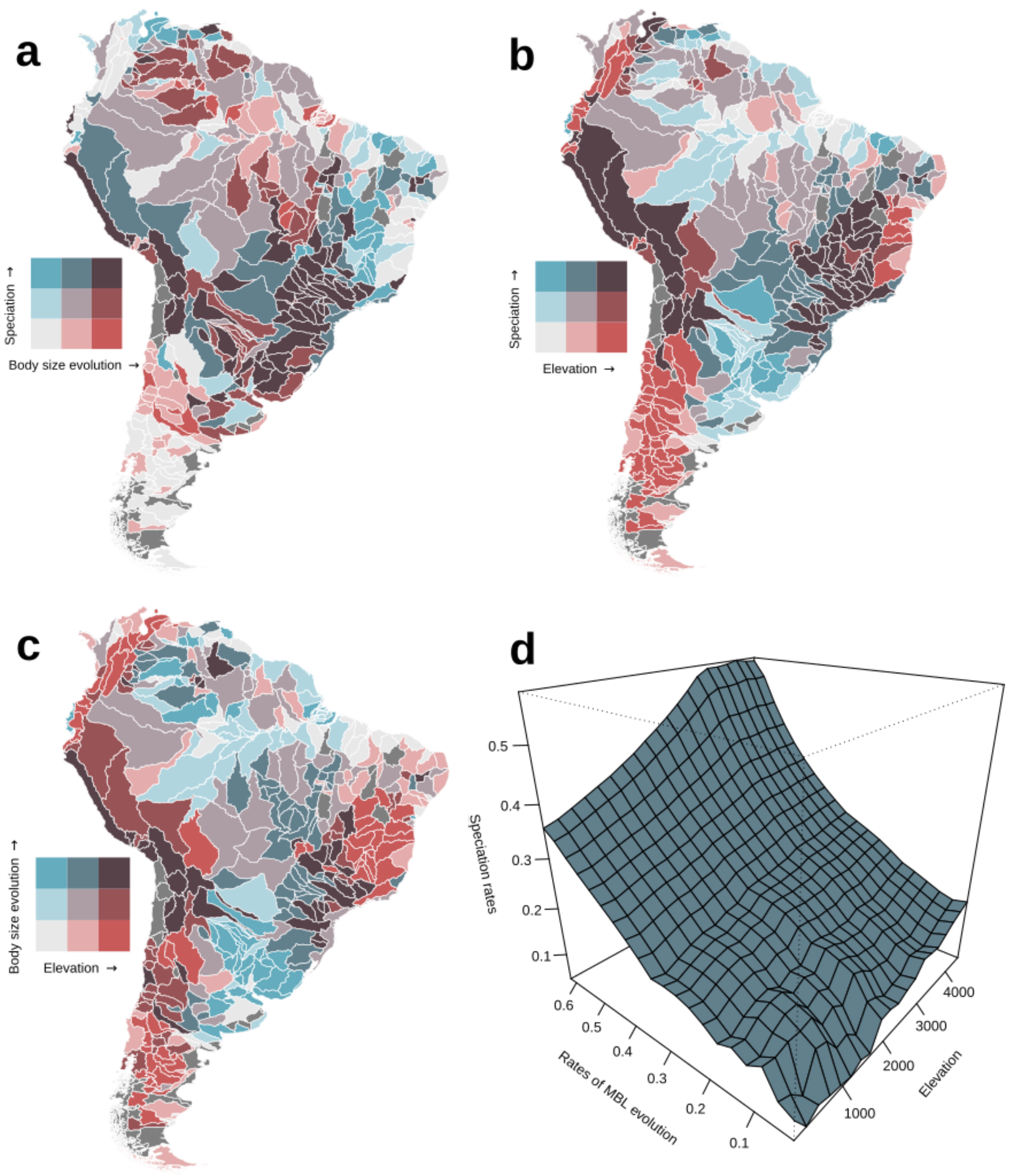

Figure S2. South American regions where speciation rates, body size evolution, and elevation are well-matched or mismatched. a Spatial covariation between rates of speciation and body size evolution. **b** Spatial covariation between rates of speciation and elevation. **c** Spatial covariation between rates of body size evolution and elevation. **d** 3D plot showing the association between elevated regions and higher rates of body size evolution, which is further associated with increased speciation rates.

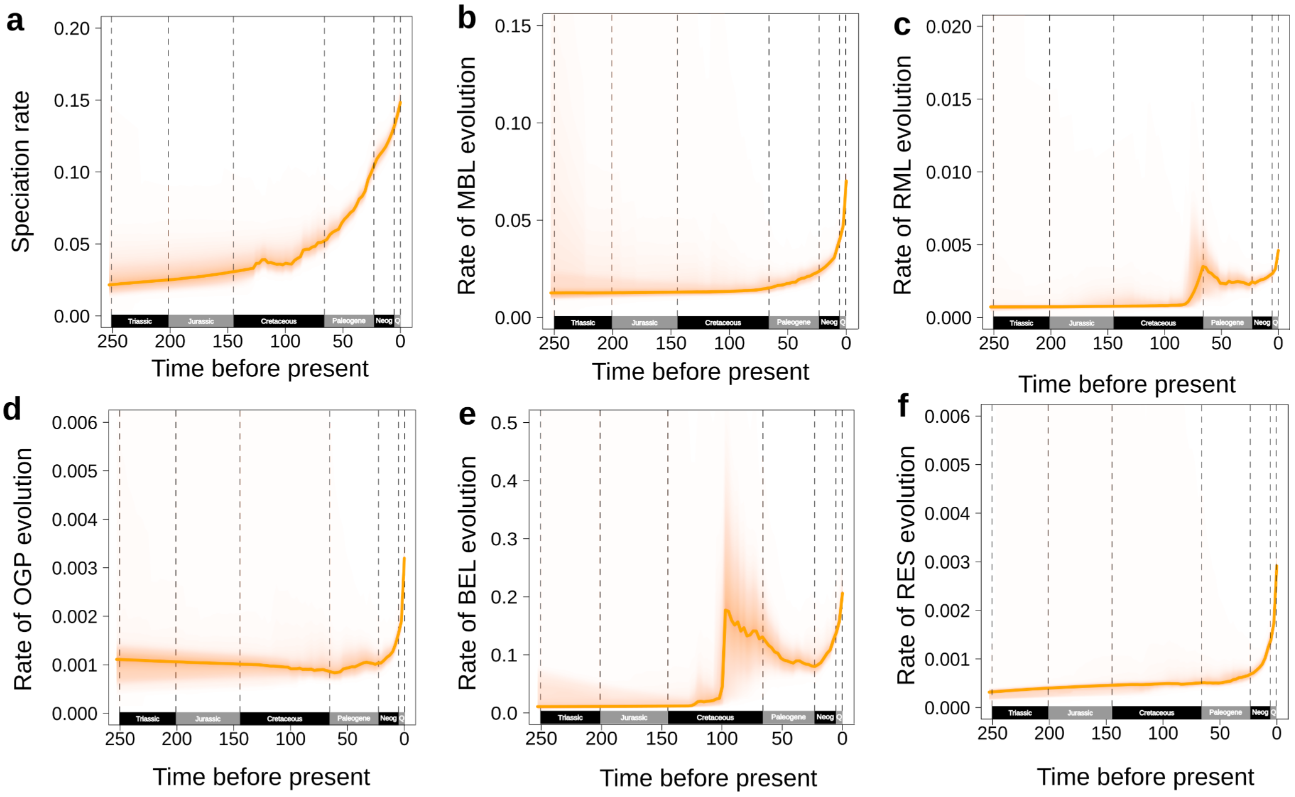

Figure S3. Temporal dynamics of evolutionary rates in South America freshwater fishes, based on BAMM analysis. The graph displays the rates-through-time pattern with corresponding confident intervals for **a** speciation rates, **b** rates of maximum body length (MBL) evolution, **c** rates of relative maxillary length (RML) evolution, **d** rates of oral gape position (OGP) evolution, **e** rates of body elongation (BEL) evolution, and **f** rates of relative eye size (RES) evolution. The Neogene and Quaternary periods are abbreviated as 'Neog' and 'Q', respectively.

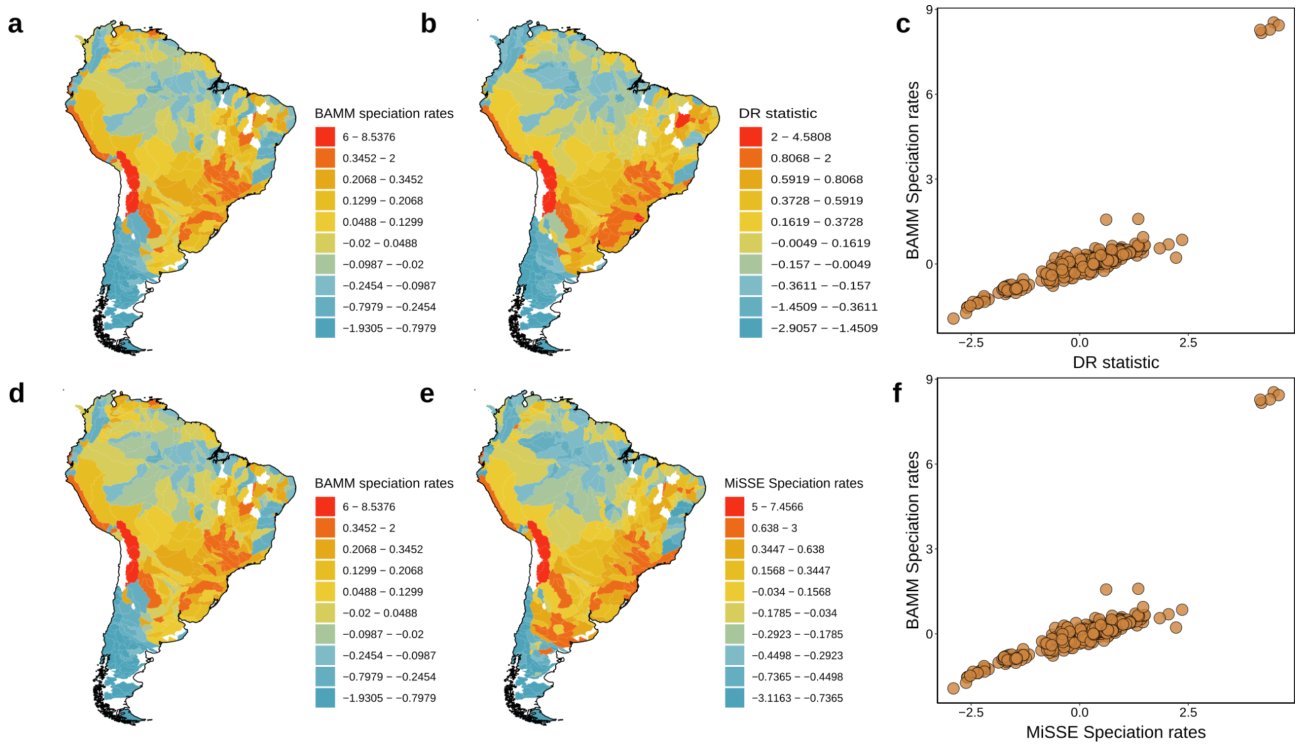

Figure S4. Geographical dynamics of speciation rates using three alternative methods. a BAMM tip speciation rate averaged in each sub-basin. **b** DR statistic estimates averaged in each sub-basin. **c** Correlation between speciation rates estimated from BAMM and DR methods ($r = 0.801$, $p < 0.001$). **d** BAMM tip speciation rate averaged in each sub-basin. **e** MiSSE tip speciation averaged in each sub-basin. **f** Correlation between speciation rates estimated from BAMM and MiSSE methods ($r = 0.917$, $p < 0.001$). Faster speciation rates are represented by red colors, while slower rates are indicated by blue colors. The speciation estimates were standardized (z-score) to facilitate comparisons between metrics.

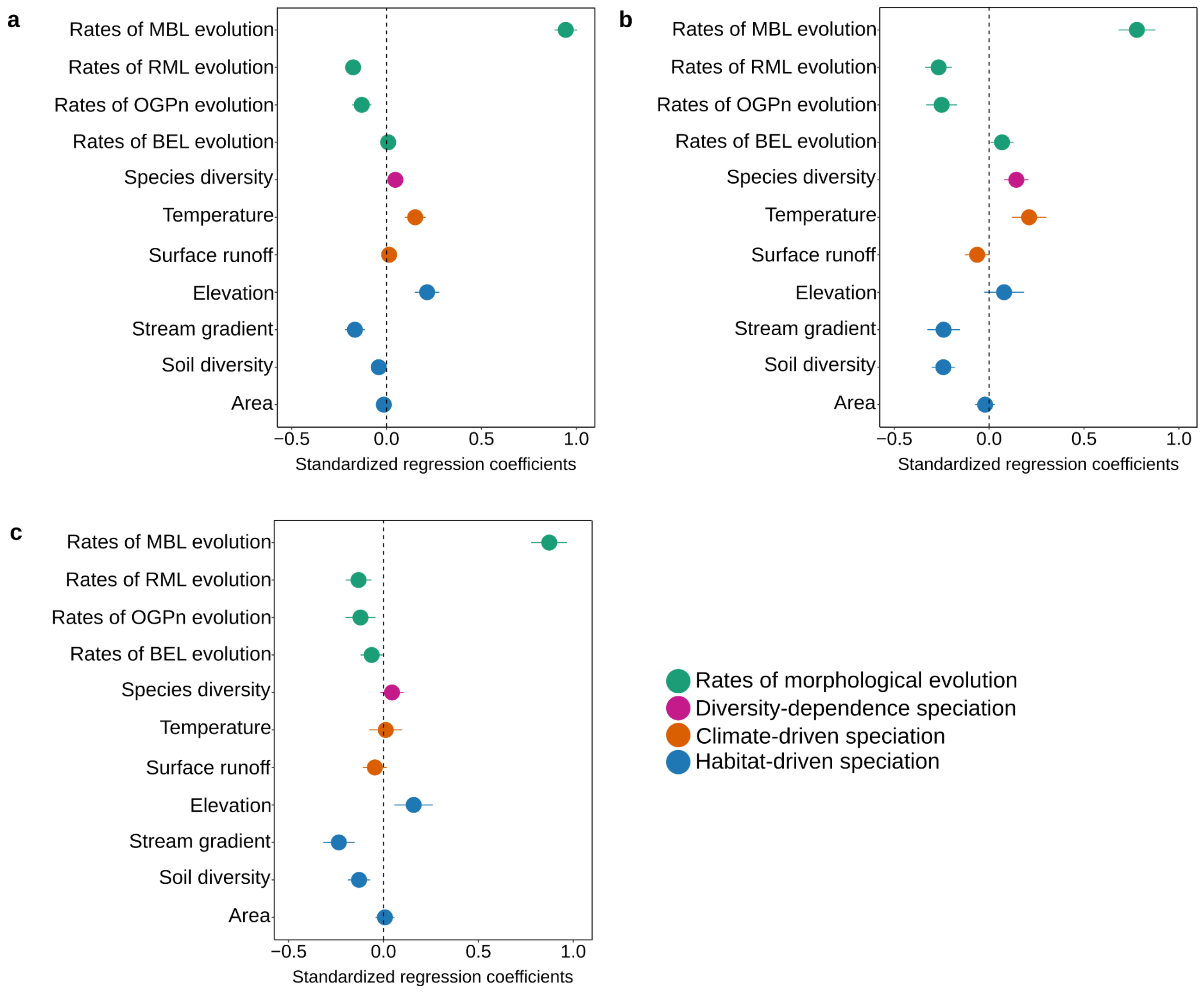

Figure S5. The relative importance of eleven biotic and abiotic factors using three speciation metrics. a Effects of multiple predictors on speciation rates measured by BAMM. **b** Effects of multiple predictors on speciation rates measured by DR statistic. **c** Effects of multiple predictors on speciation rates measured by MiSSE. Standardized regression coefficients and their 95% confidence intervals are displayed for each predictor. Abbreviations for rates of morphological evolution: body elongation (BEL), maximum body length (MBL), oral gape position (OGP), and relative maxillary length (RML).

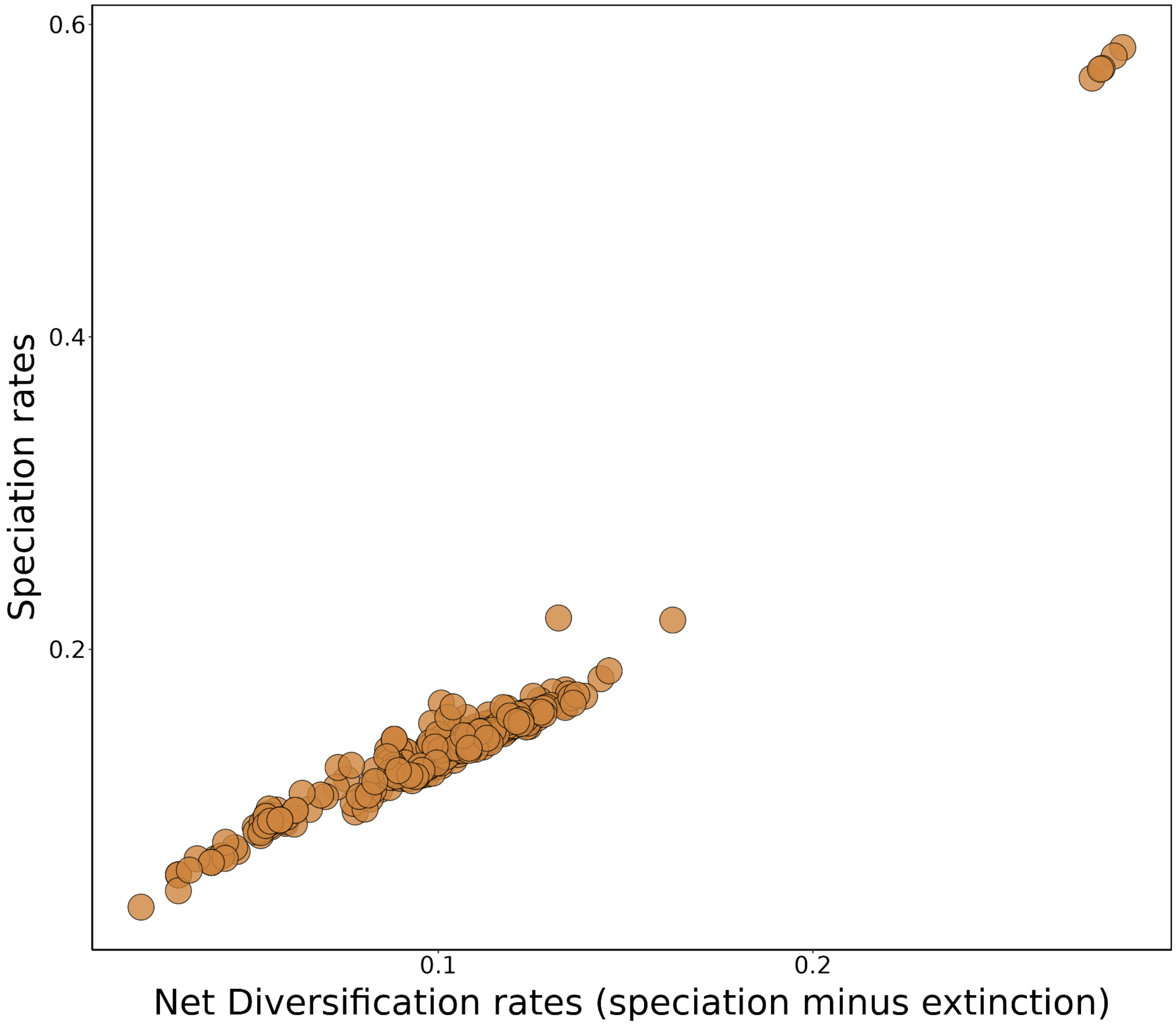

Figure S6. Correlation between BAMM speciation rates and BAMM net diversification rates in South American freshwater fish species. We compared the BAMM speciation rates and BAMM net diversification rates (speciation minus extinction) estimated from tip-based analyses and found a strong positive correlation between them (Pearson's $r = 0.914$, $p < 0.001$).

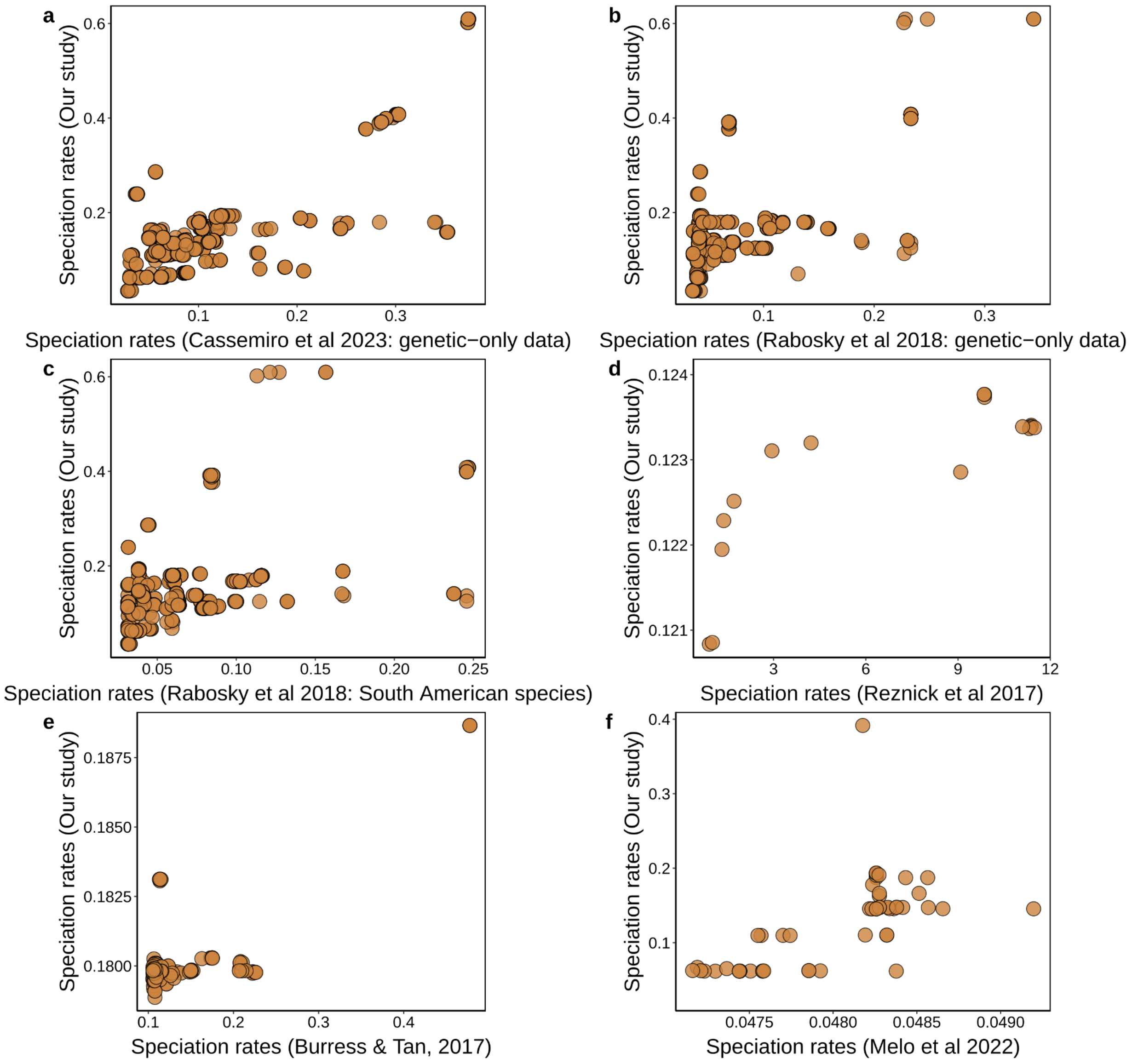

Figure S7. Comparison of BAMM tip-based speciation rates estimates across phylogenies of South American freshwater fish species. We evaluated the effects of phylogenetic uncertainties on the BAMM speciation rate estimates by comparing the rates estimated from the taxonomic-genetic tree of Cassemiro et al., 2023 (our study) with those estimated from other published trees. The scatterplots show the correlation between the speciation rates estimated from our tree and those estimated from other trees: **a** Cassemiro et al., 2023 tree with genetic-only data (Pearson's r = 0.822, p < 0.001), **b** Rabosky et al., 2018 tree with genetic-only data (r = 0.637, p < 0.001), **c** Rabosky et al., 2018 tree including South American species with genetic-only data (r = 0.608, p < 0.001), **d** Reznick *et al.*, 2017 tree (r = 0.803, p < 0.001), **e** Burress & Tan, 2017 tree (r = 0.633, p < 0.001), and **f** Melo et al., 2022 tree (r = 0.655, p < 0.001). The variation in y-axis values across panels occurs because different sets of species are utilized to align with other phylogenetic frameworks. Each dot represents the BAMM tip speciation rates.

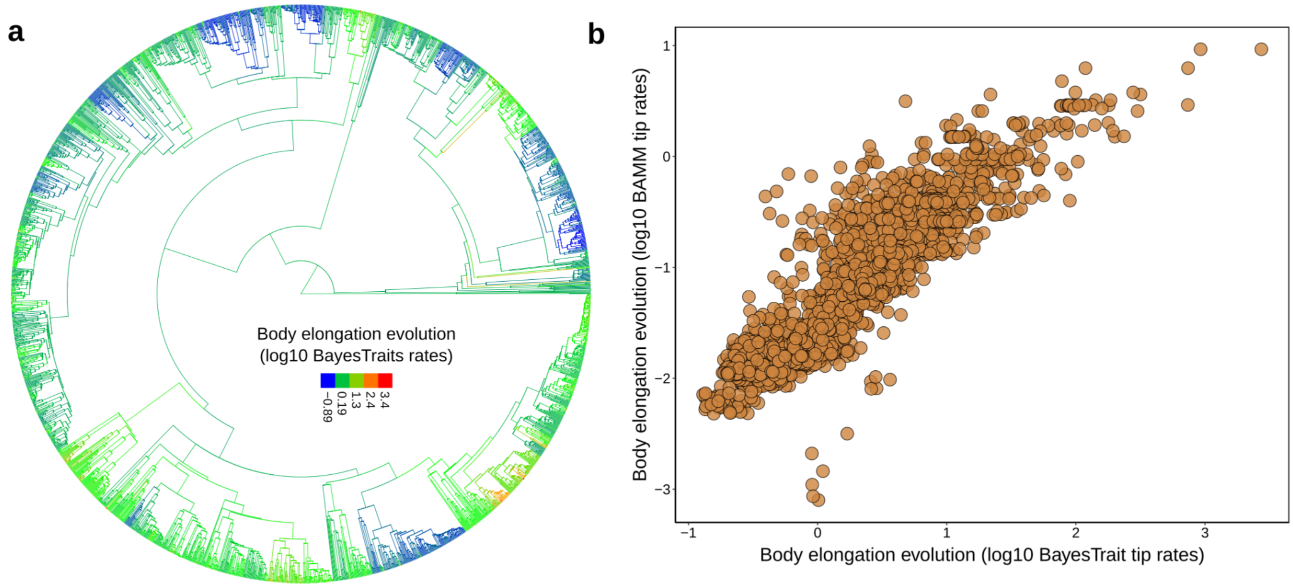

Figure S8. Comparison of body elongation (BEL) evolution rates estimated using BayesTraits and BAMM methods in a phylogeny of 2,638 South American freshwater fish species. a Estimated rates of BEL evolution using BayesTraits method, with branches colored to represent slower rates (blue and green) and faster rates (orange and red). **b** Scatter plot illustrating the relationship between BEL evolution rates estimated by BayesTraits and BAMM methods (Pearson's $r = 0.900$, $p < 0.001$). Each dot represents a tip-based estimate of BEL evolution for a particular fish species.

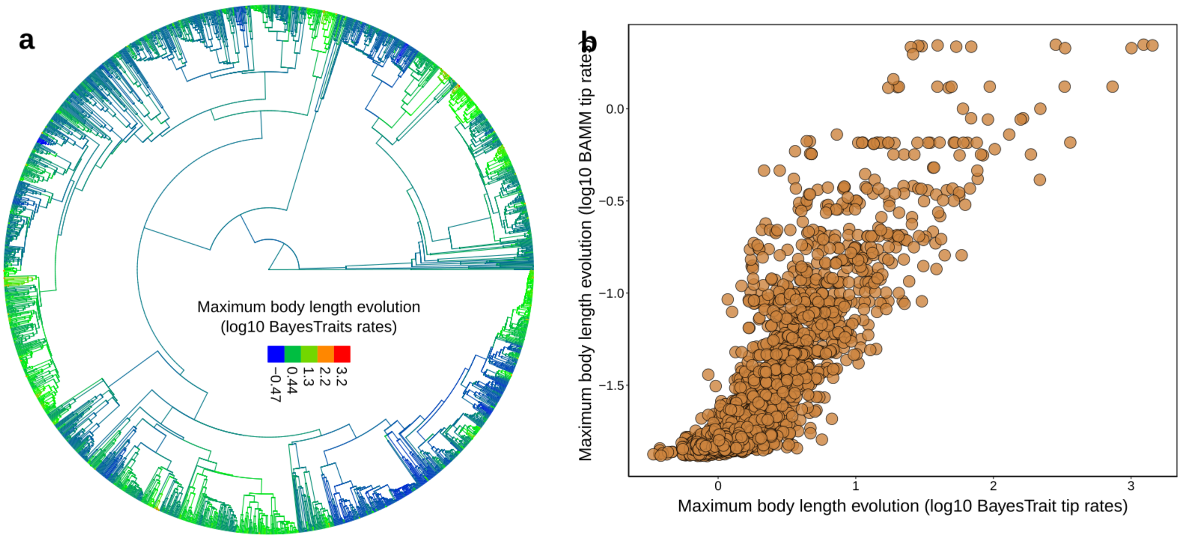

Figure S9. Comparison of maximum body length (MBL) evolution rates estimated using BayesTraits and BAMM methods in a phylogeny of 2,638 South American freshwater fish species. a Estimated rates of MBL evolution using BayesTraits method, with branches colored to represent slower rates (blue and green) and faster rates (orange and red). **b** Scatter plot illustrating the relationship between MBL evolution rates estimated by BayesTraits and BAMM methods (Pearson's $r = 0.870$, $p < 0.001$). Each dot represents a tip-based estimate of BEL evolution for a particular fish species.

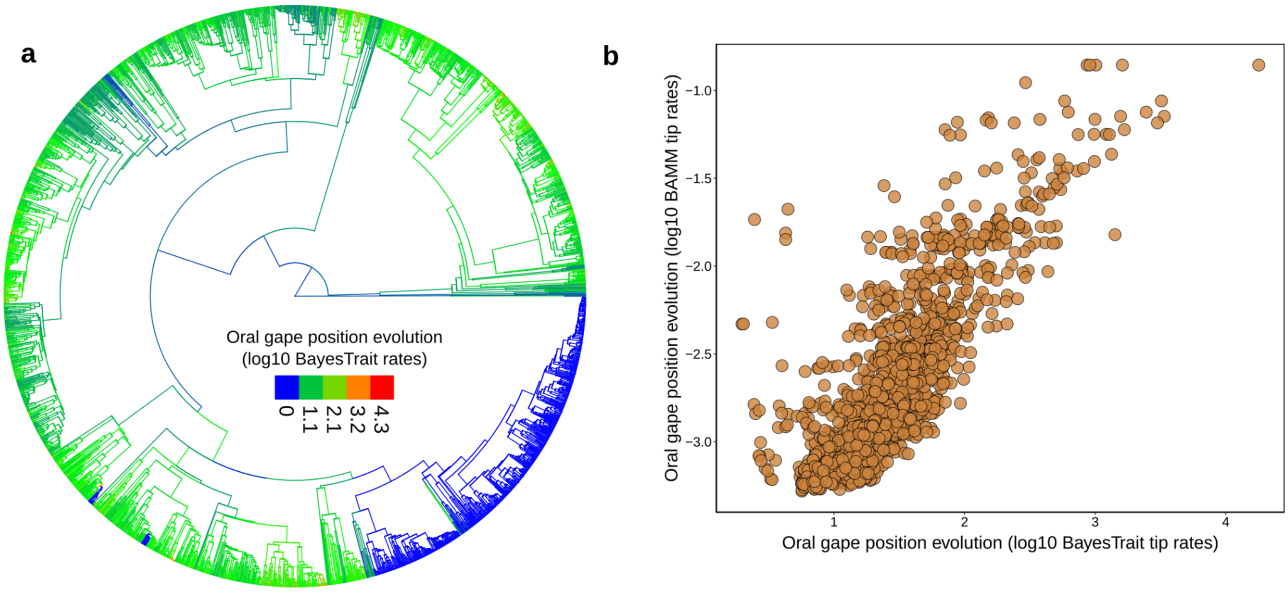

Figure S10. Comparison of oral gape position (OGP) evolution rates estimated using BayesTraits and BAMM methods in a phylogeny of 2,638 South American freshwater fish species. a Estimated rates of OGP evolution using BayesTraits method, with branches colored to represent slower rates (blue and green) and faster rates (orange and red). **b** Scatter plot illustrating the relationship between OGP evolution rates estimated by BayesTraits and BAMM methods (Pearson's $r = 0.878$, $p < 0.001$). Each dot represents a tip-based estimate of OGP evolution for a particular fish species.

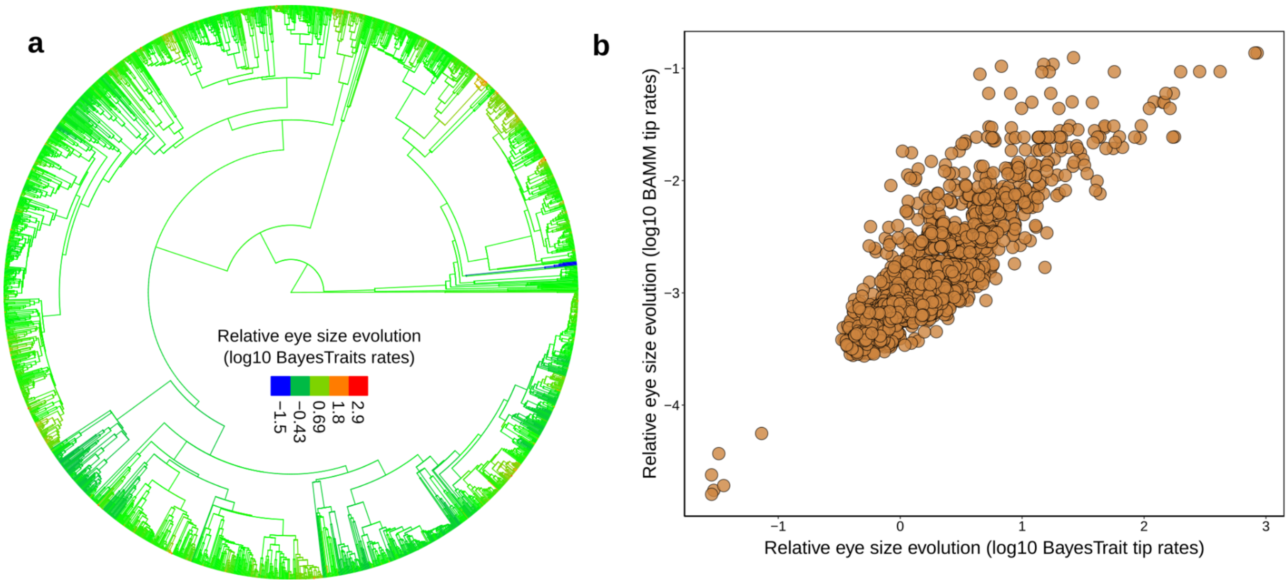

Figure S11. Comparison of relative eye size (RES) evolution rates estimated using BayesTraits and BAMM methods in a phylogeny of 2,638 South American freshwater fish species. a Estimated rates of RES evolution using BayesTraits method, with branches colored to represent slower rates (blue and green) and faster rates (orange and red). **b** Scatter plot illustrating the relationship between RES evolution rates estimated by BayesTraits and BAMM methods (Pearson's $r = 0.873$, $p < 0.001$). Each dot represents a tip-based estimate of RES evolution for a particular fish species.

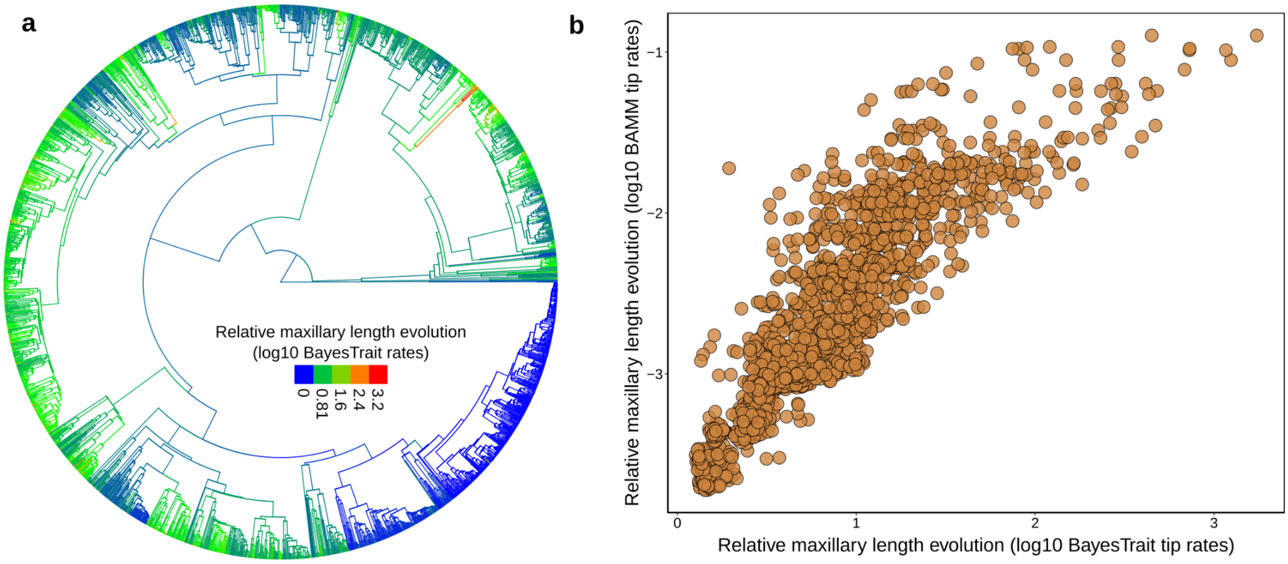

Figure S12. Comparison of relative maxillary length (RML) evolution rates estimated using BayesTraits and BAMM methods in a phylogeny of 2,638 South American freshwater fish species. a Estimated rates of RML evolution using BayesTraits method, with branches colored to represent slower rates (blue and green) and faster rates (orange and red). **b** Scatter plot illustrating the relationship between RML evolution rates estimated by BayesTraits and BAMM methods (Pearson's $r = 0.889$, $p < 0.001$). Each dot represents a tip-based estimate of RML evolution for a particular fish species.

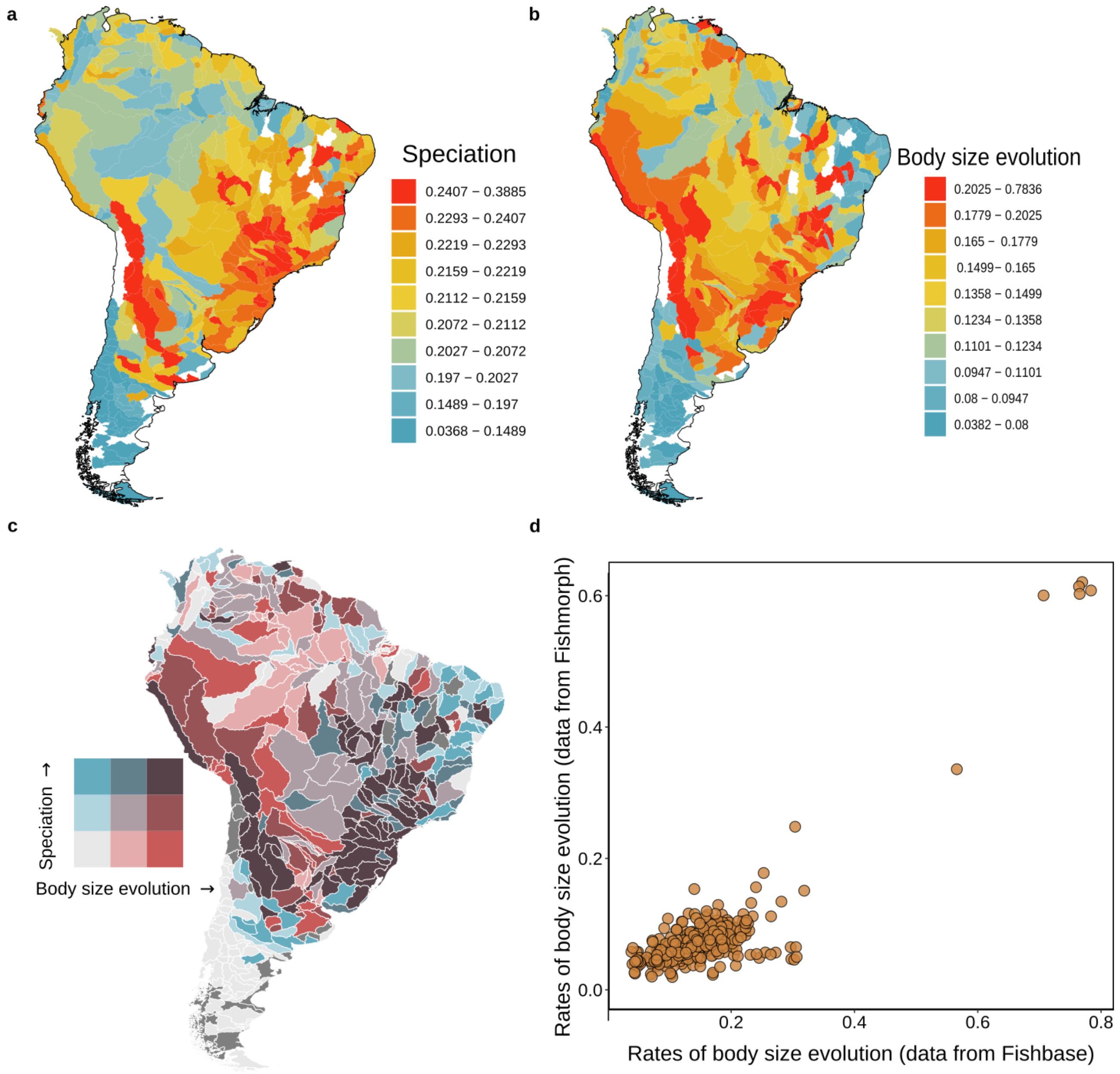

Figure S13. The impact of incomplete taxonomic coverage on estimates of evolutionary rates. a BAMM tip speciation rates averaged in each sub-basin based on 4,228 species. **b** Rates of maximum body length (MBL) evolution measured in each sub-basin based on 4,228 species. **c** Spatial matches and mismatches between rates of speciation and body size evolution (**a** and **b** panels). **d** Correlation between rates of body size evolution across sub-basins estimated from our study (2,638 species with data from fishmorph) and data from Fishbase (4,228 species) (Pearson's $r = 0.870$, $p < 0.001$). Red colors indicate faster and blue colors indicate slower rates. Evolutionary rates in **a**, **b**, and **c** were estimated using a phylogeny including 4,228 species (85% of the South American fauna) and data from Fishbase on maximum body length.

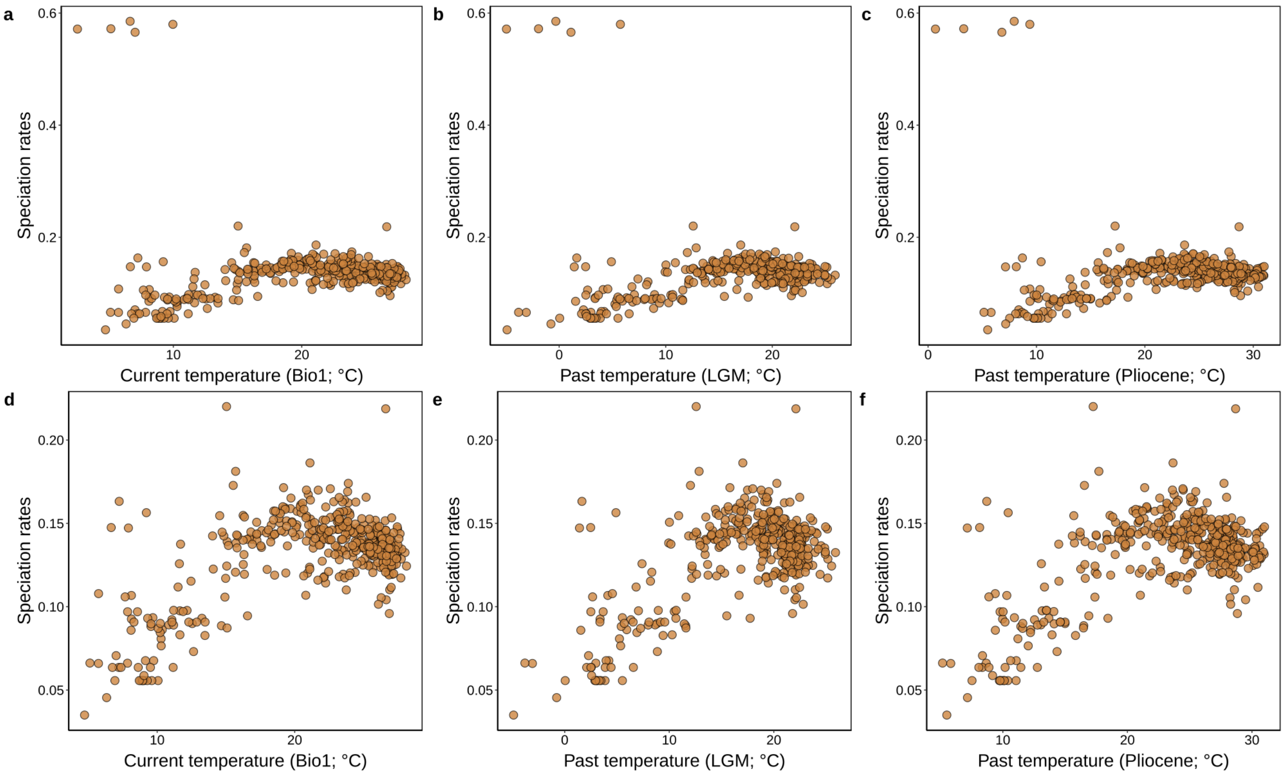

Figure S14. Effects of past temperature on speciation rates. Relationships between speciation rates and **a** current temperature (Pearson's r = 0.060, p = 0.195), **b** Last Glacial Maximum temperature (ca. 21 ka) (r = 0.088, p = 0.057), **c** Pliocene temperature (ca. 3.3 My) (r = 0.102, p = 0.027), **d** current temperature excluding outliers (r = 0.093, p = 0.047), **e** Last Glacial Maximum temperature excluding outliers (r = 0.122, p = 0.009), and **f** Pliocene temperature excluding outliers (r = 0.138, p = 0.003). Points indicate estimates obtained for each South American sub-basin. The lower panels depict the temperature-speciation relationship with outliers removed. Five sub-basins predominantly inhabited by Orestias species were considered outliers. Interestingly, the effects of past temperature on speciation rates align with current temperature observations.

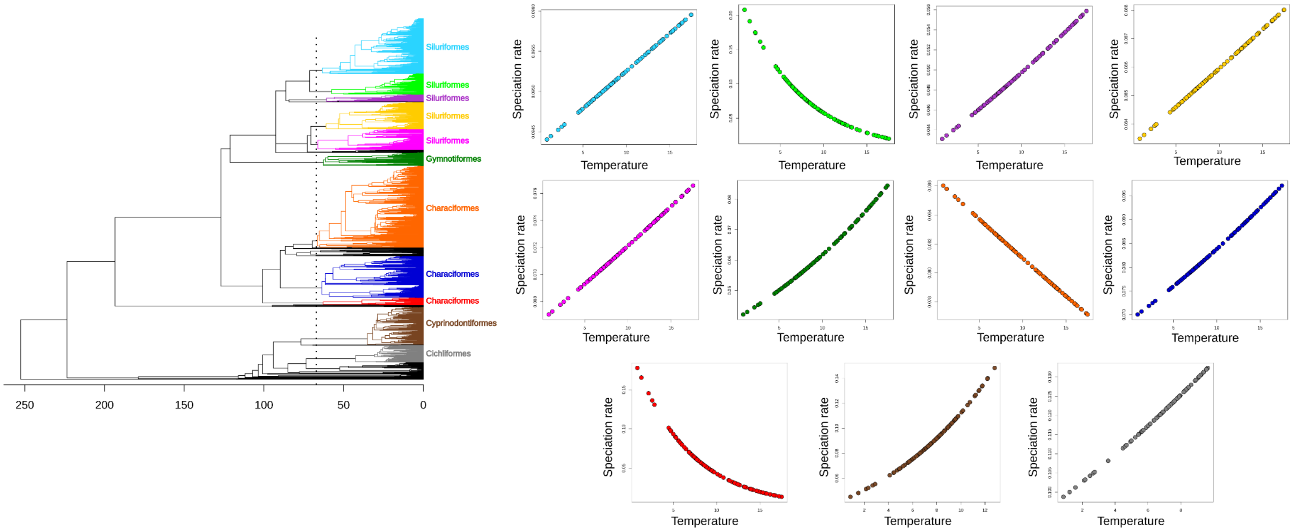

Figure S15. Temperature-dependent speciation model as calculated by RPANDA. Paleotemperature was based on Zachos et al., 2008, covering historical temperature data through the Cenozoic period. We selected 11 clades with more than 50 species that are within this time frame, which are highlighted by the dashed line and different colors in the phylogeny. The scatterplot represents speciation rates as a function of temperature, with scatterplot colors corresponding to the same colors of the clades in the phylogeny. Notably, eight out of eleven clades exhibit a positive correlation between speciation rates and temperature.

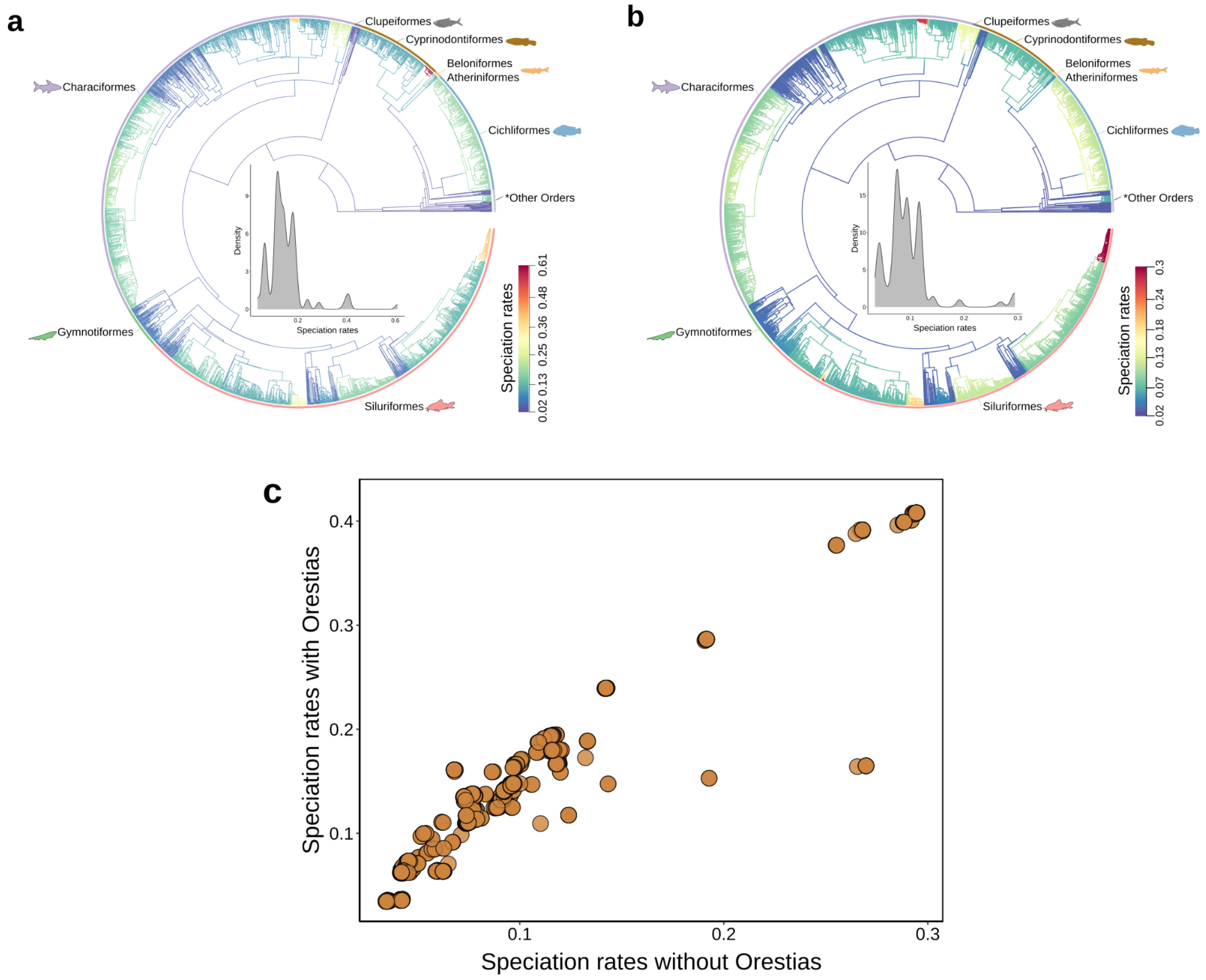

Figure S16. Dynamics of speciation rates across the phylogeny. Speciation rates were estimated using Bayesian analysis of macroevolutionary mixtures (BAMM). **a** BAMM speciation rates with *Orestias* species included. **b** BAMM speciation rates with *Orestias* species removed prior to the analysis. Branches were colored to represent slower (blue and green) and faster (orange and red) speciation rates. **c** Correlation between tip-based rates of speciation with or without *Orestias* species during BAMM analysis (Pearson's r = 0.965, p < 0.001). Note that BAMM speciation tip-rates were consistent with or without *Orestias* species, as evidenced by the similar shape of the density plots. This suggests that the exceptionally high speciation observed in this small clade does not obscure the chances of detecting high rates in other clades.

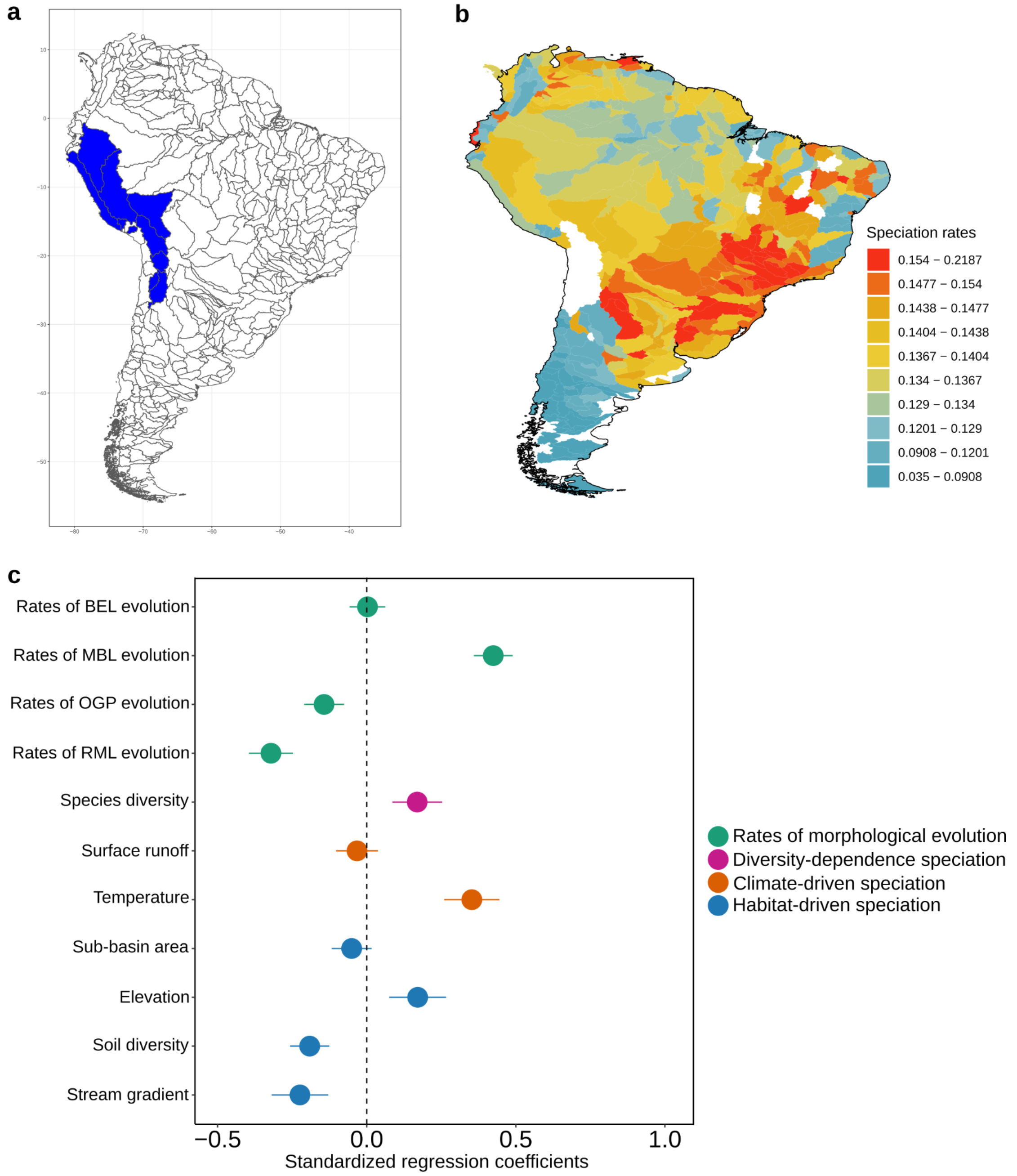

Figure S17. Impact of *Orestias* species removal on speciation rate. a Sub-basins highlighted in blue indicate those sub-basins where at least one *Orestias* species occurs. **b** Mean speciation rate pattern without the presence of *Orestias* species in any sub-basin. Sub-basins are colored to represent slower (blue and green) and faster (orange and red) rates. **c** Standardized regression coefficients with 95% confidence intervals are shown for each predictor after removing all *Orestias* species from the mean speciation rates across sub-basins. Abbreviations for rates of morphological evolution: body elongation (BEL), maximum body length (MBL), oral gape position (OGP), and relative maxillary length (RML).

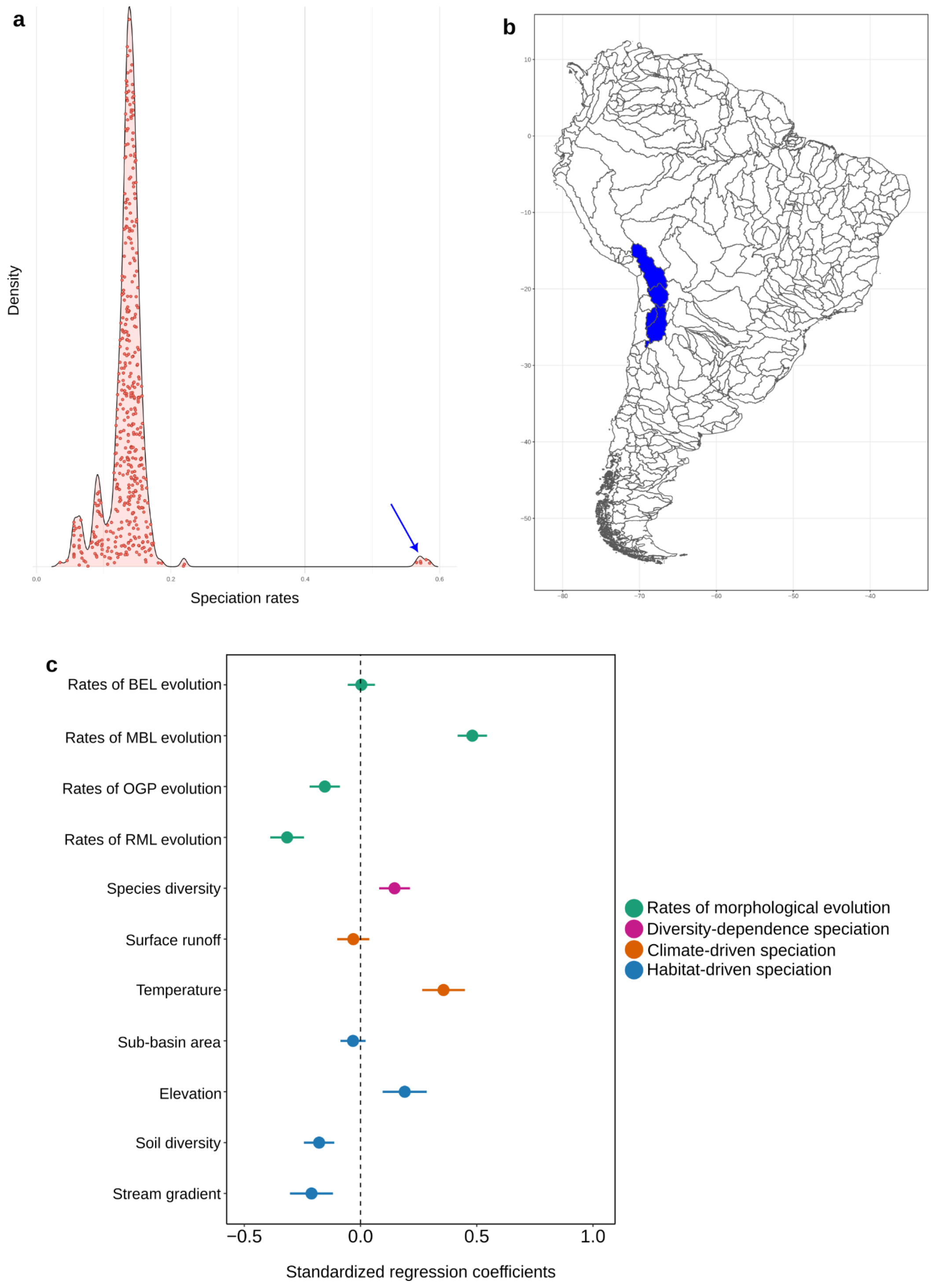

Figure S18. Five sub-basins recovered by our analyses as biological outliers. These sub-basins are predominantly occupied by *Orestias* species. **a** The distribution of speciation rates estimates across South American sub-basins. The blue arrow indicates the five sub-basins with exceptional speciation rates. **b** Map highlighting the referred sub-basins. **c** Standardized regression coefficients with 95% confidence intervals are shown for each predictor after removing the five sub-basins. Abbreviations for rates of morphological evolution: body elongation (BEL), maximum body length (MBL), oral gape position (OGP), and relative maxillary length (RML).

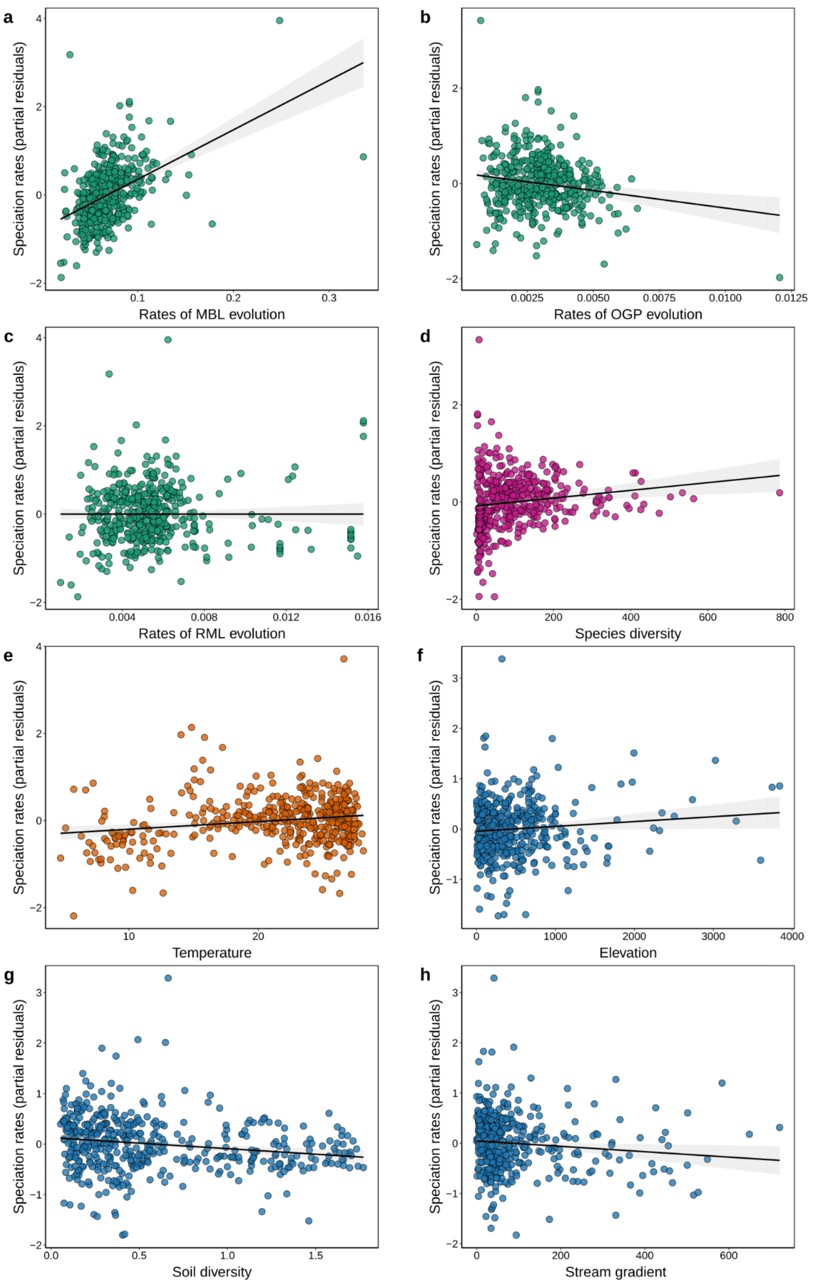

Figure S19. Partial residual plots depicting the relationship between speciation rates and biotic and abiotic variables after removing five outliers sub-basins (Supplementary Figure 18b). Partial residuals plots were obtained from a multiple linear regression of the relationship between speciation rates and **a** rates of maximum body length (MBL) evolution, **b** rates of oral gape position (OGP) evolution, **c** rates of relative maxillary length (RML) evolution, **d** species diversity, **e** temperature, **f** elevation, **g** soil diversity, **h** stream gradient. The colors represent the four main mechanisms (Table 1): rates of morphological evolution (green), diversity-dependence (pink) climate-driven (orange), and habitat-driven (blue). Compare with Figure 2 to see qualitatively similar results and that these five outliers do not skew our conclusions.

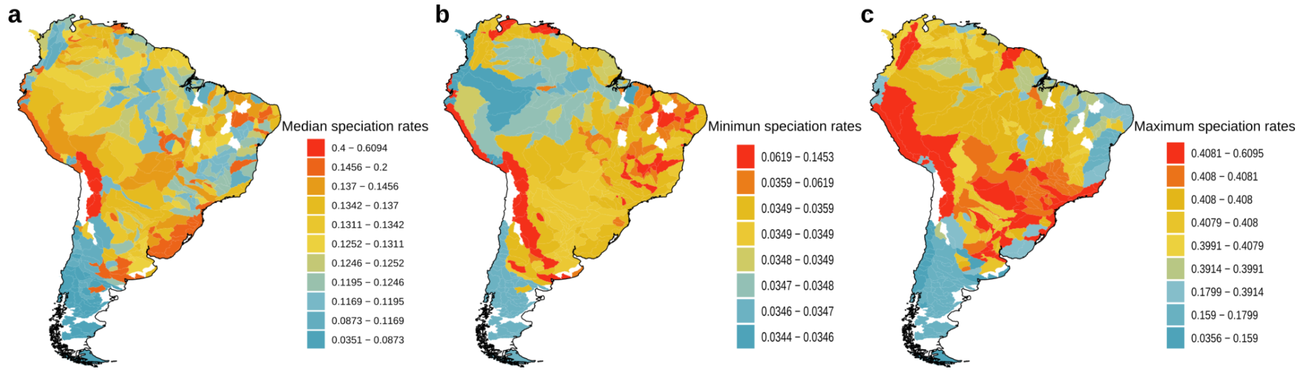

Figure S20. Spatial variation in speciation rates using different summary statistics. a Median speciation rates for each sub-basin. **b** Minimum speciation rates for each sub-basin. **c** Maximum speciation rates for each sub-basin. The color scale ranges from blue (slower speciation rates) to red (faster speciation rates).

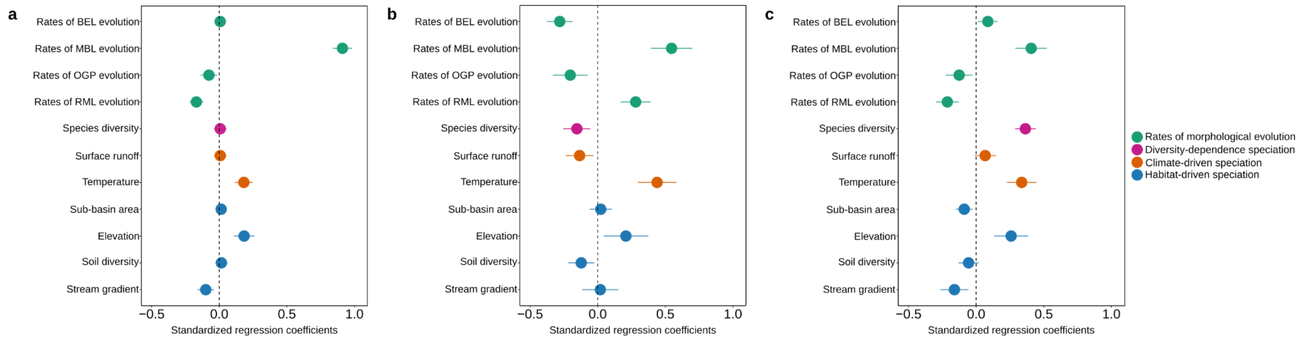

Figure S21. Influence of various drivers on speciation rates using different summary statistics. a Standardized regression coefficients with 95% confidence intervals are displayed for each predictor on median speciation rates. **b** Standardized regression coefficients with 95% confidence intervals are displayed for each predictor on minimum speciation rates. **c** Standardized regression coefficients with 95% confidence intervals are displayed for each predictor on maximum speciation rates. Abbreviations for rates of morphological evolution: body elongation (BEL), maximum body length (MBL), oral gape position (OGP), and relative maxillary length (RML).

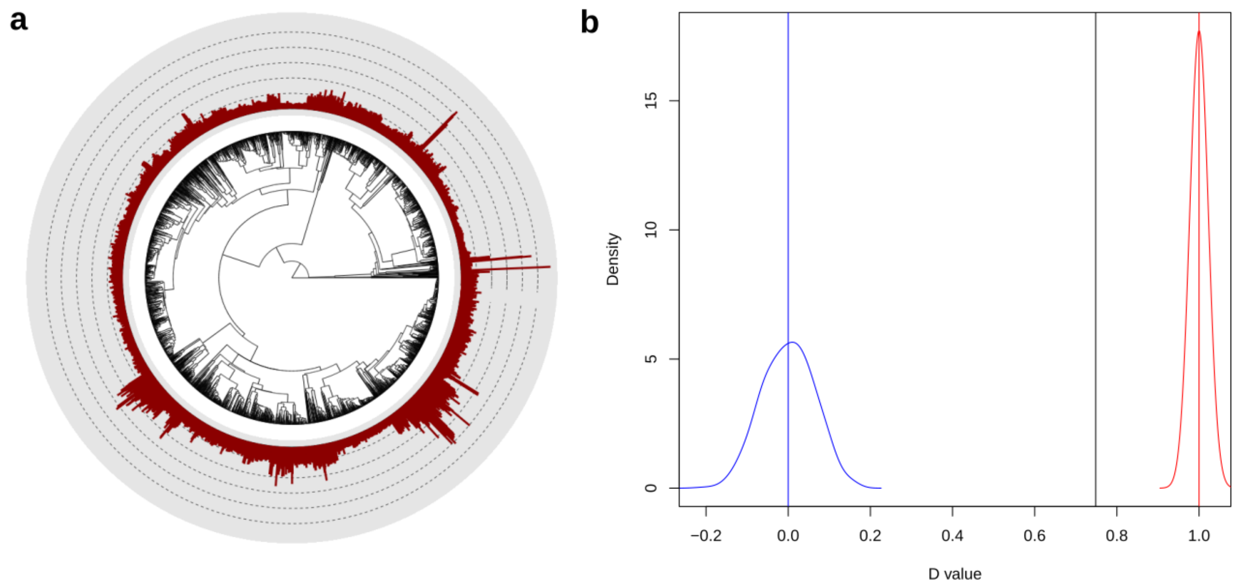

Figure S22. Distribution of body elongation (BEL) across the phylogeny and assessment of phylogenetic signal in missing data. a Distribution of raw BEL data across the phylogeny, which includes 2,638 species. **b** D-statistic estimates for phylogenetic signal in missing BEL data (0.6%). The black line represents the estimated D value (D = 0.75), which was significantly different from what would be expected in the case of highly conserved (blue line; $p < 0.05$) or overdispersed (red line; $p < 0.05$) missing data.

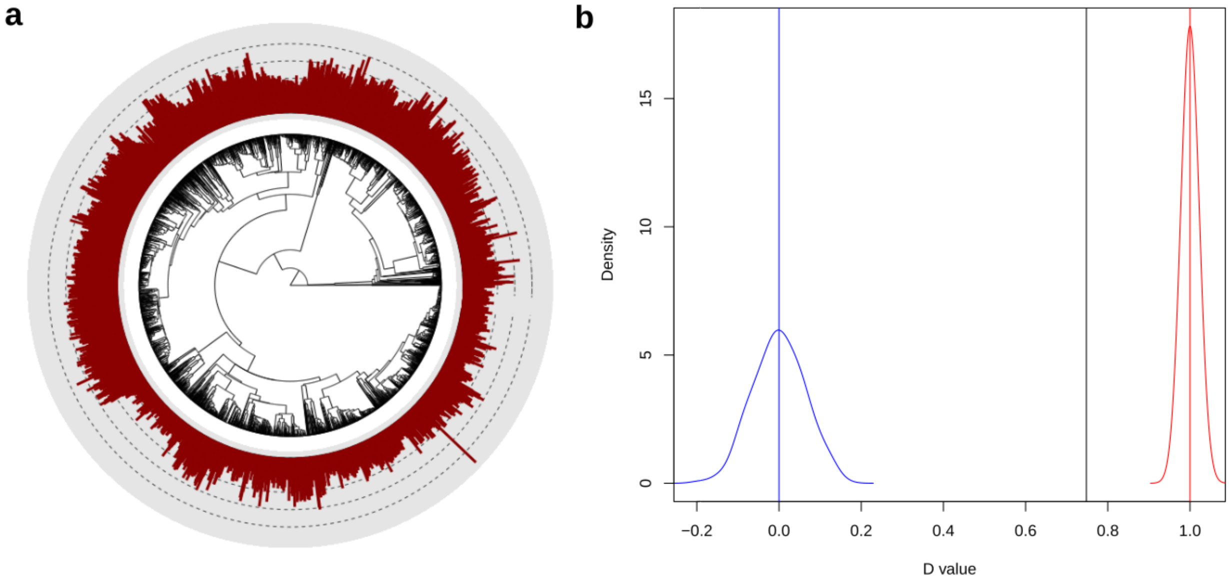

Figure S23. Distribution of relative eye size (RES) across the phylogeny and assessment of phylogenetic signal in missing data. a Distribution of raw RES data across the phylogeny, which includes 2,638 species. **b** D-statistic estimates for phylogenetic signal in missing RES data (0.6%). The black line represents the estimated D value (D = 0.75), which was significantly different from what would be expected in the case of highly conserved (blue line; $p < 0.05$) or overdispersed (red line; $p < 0.05$) missing data.

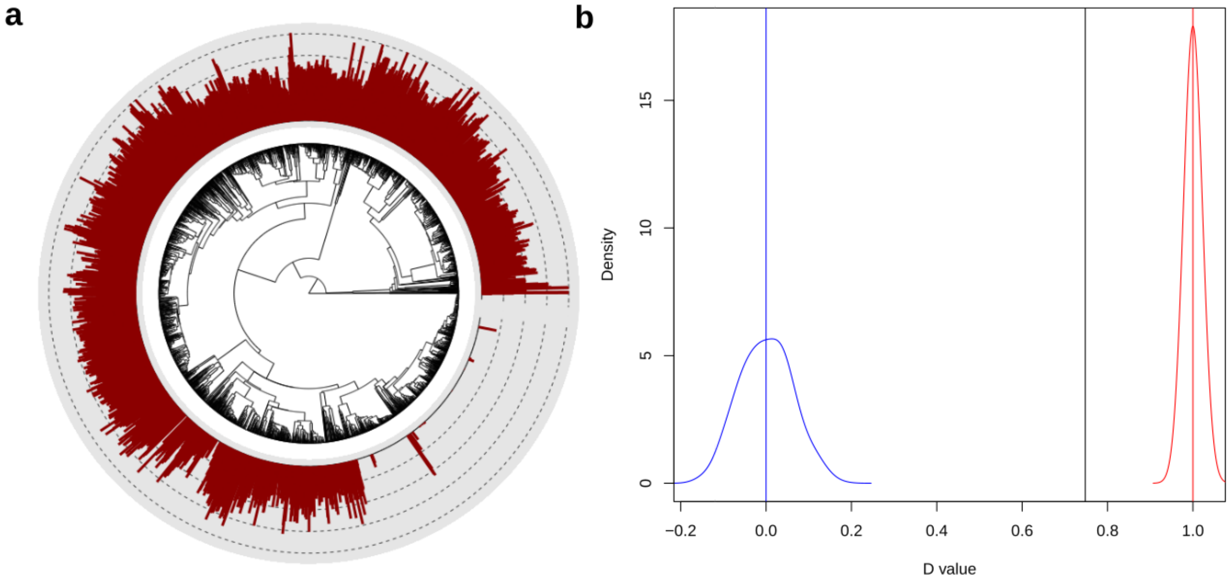

Figure S24. Distribution of oral gape position (OGP) across the phylogeny and assessment of phylogenetic signal in missing data. a Distribution of raw OGP data across the phylogeny, which includes 2,638 species. **b** D-statistic estimates for phylogenetic signal in missing OGP data (0.6%). The black line represents the estimated D value (D = 0.75), which was significantly different from what would be expected in the case of highly conserved (blue line; $p < 0.05$) or overdispersed (red line; $p < 0.05$) missing data.

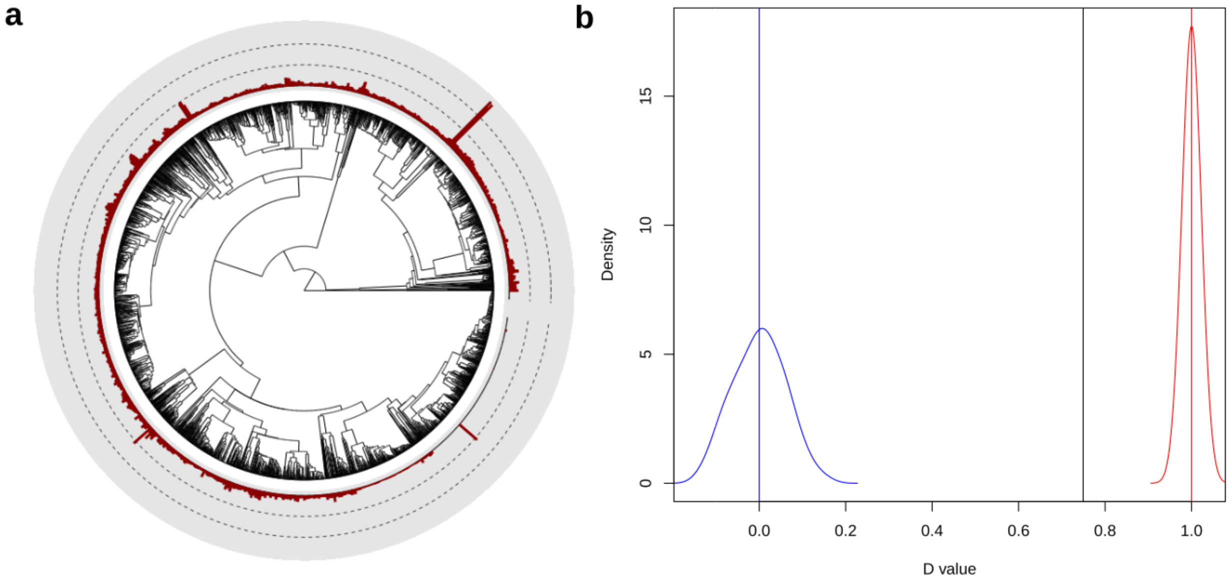

Figure S25. Distribution of relative maxillary length (RML) across the phylogeny and assessment of phylogenetic signal in missing data. **a** Distribution of raw RML data across the phylogeny, which includes 2,638 species. **b** D-statistic estimates for phylogenetic signal in missing RML data (0.6%). The black line represents the estimated D value (D = 0.75), which was significantly different from what would be expected in the case of highly conserved (blue line; $p < 0.05$) or overdispersed (red line; $p < 0.05$) missing data.

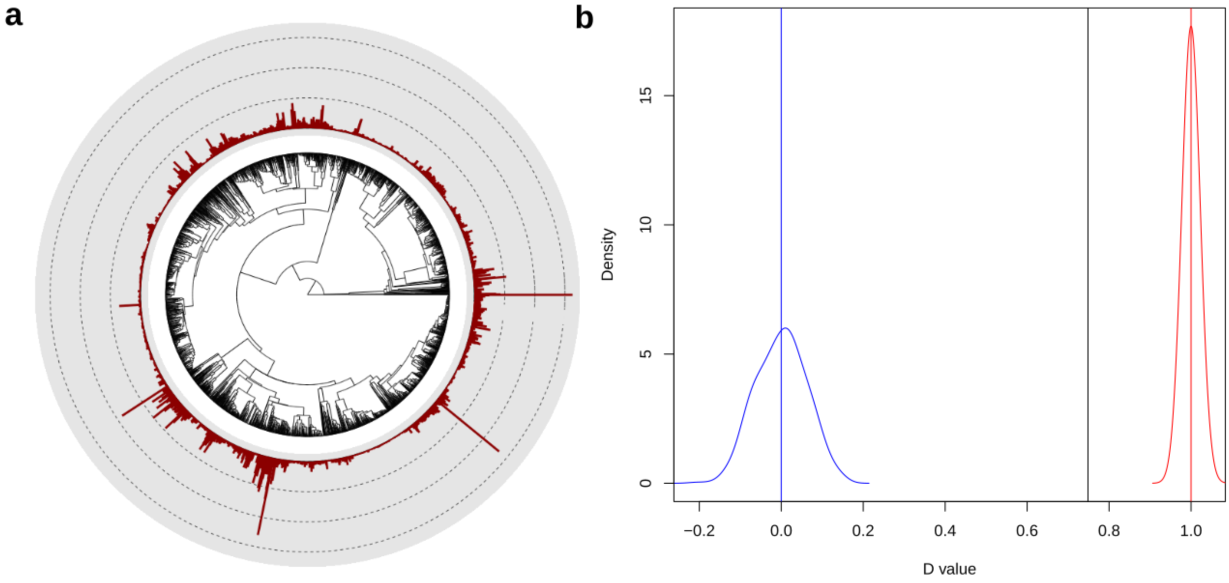

Figure S26. Distribution of maxim body length (MBL) across the phylogeny and assessment of phylogenetic signal in missing data. a Distribution of raw MBL data across the phylogeny, which includes 2,638 species. **b** D-statistic estimates for phylogenetic signal in missing MBL data (0.6%). The black line represents the estimated D value (D = 0.75), which was significantly different from what would be expected in the case of highly conserved (blue line; $p < 0.05$) or overdispersed (red line; $p < 0.05$) missing data.

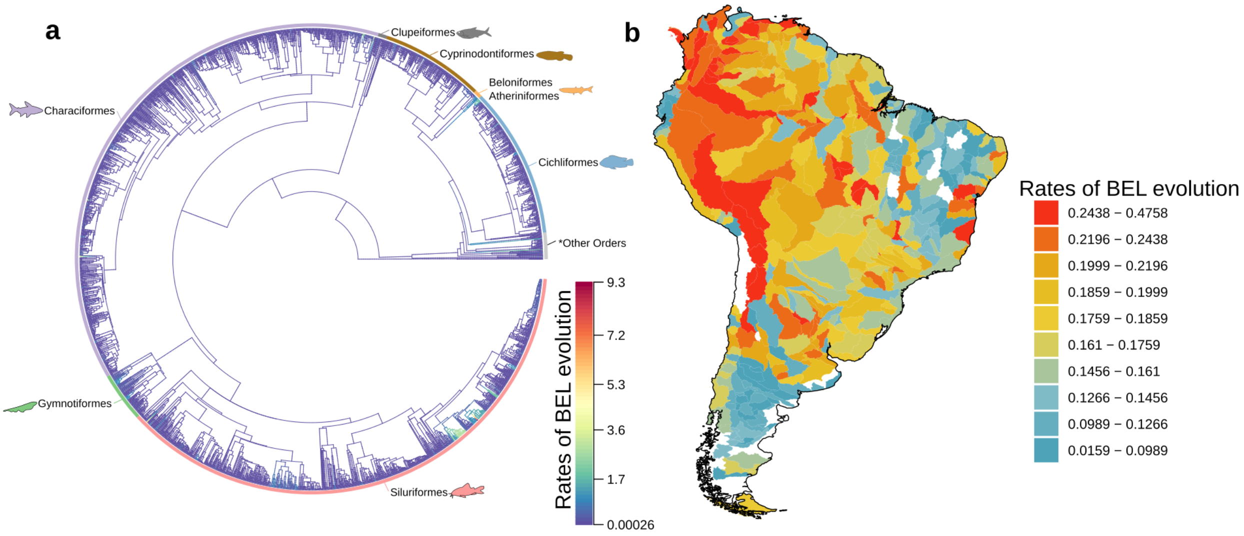

Figure S27. Temporal and geographic patterns of morphological evolution. Rates of body elongation (BEL) evolution measured across: **a** the entire phylogeny including 2,638 species, and **b** South American sub-basins. Evolutionary rates of BEL were estimated using the BAMM 'trait' module. Evolutionary rates in sub-basins were calculated by averaging BAMM tip rates of co-occurring species.

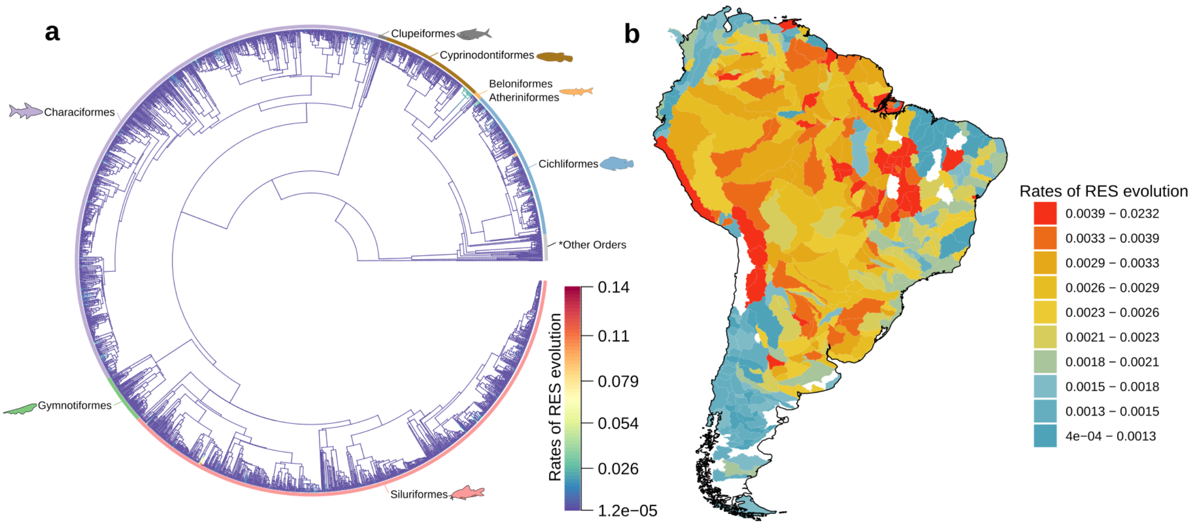

Figure S28. Temporal and geographic patterns of morphological evolution. Rates of relative eye size (RES) evolution measured across: **a** the entire phylogeny including 2,638 species, and **b** South American sub-basins. Evolutionary rates of RES were estimated using the BAMM 'trait' module. Evolutionary rates in sub-basins were calculated by averaging BAMM tip rates of co-occurring species.

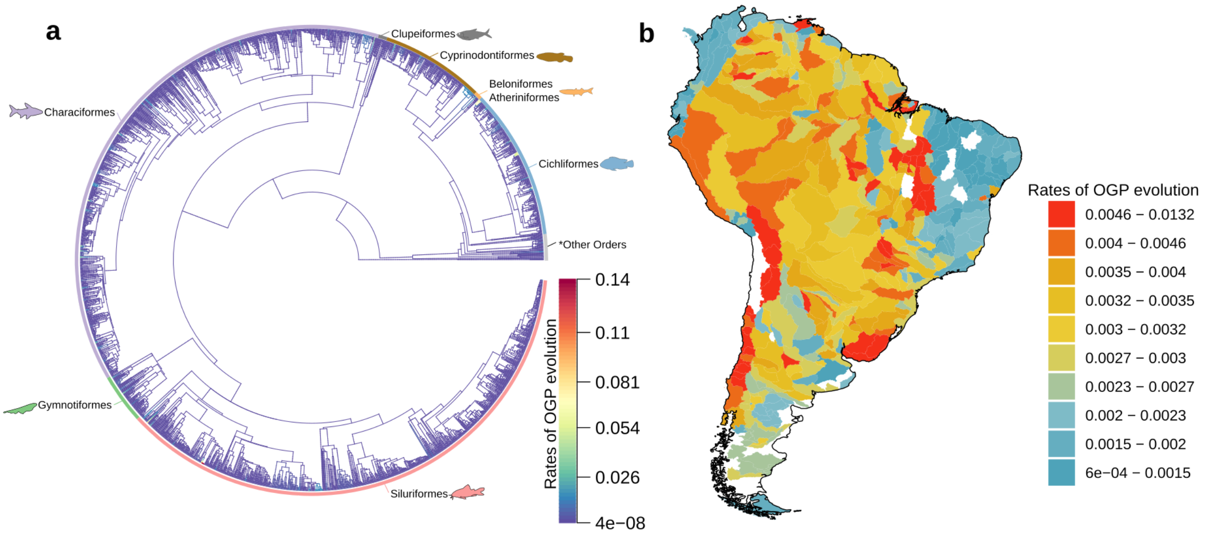

Figure S29. Temporal and geographic patterns of morphological evolution. Rates of oral gape position (OGP) evolution measured across: **a** the entire phylogeny including 2,638 species, and **b** South American sub-basins. Evolutionary rates of OGP were estimated using the BAMM 'trait' module. Evolutionary rates in sub-basins were calculated by averaging BAMM tip rates of co-occurring species.

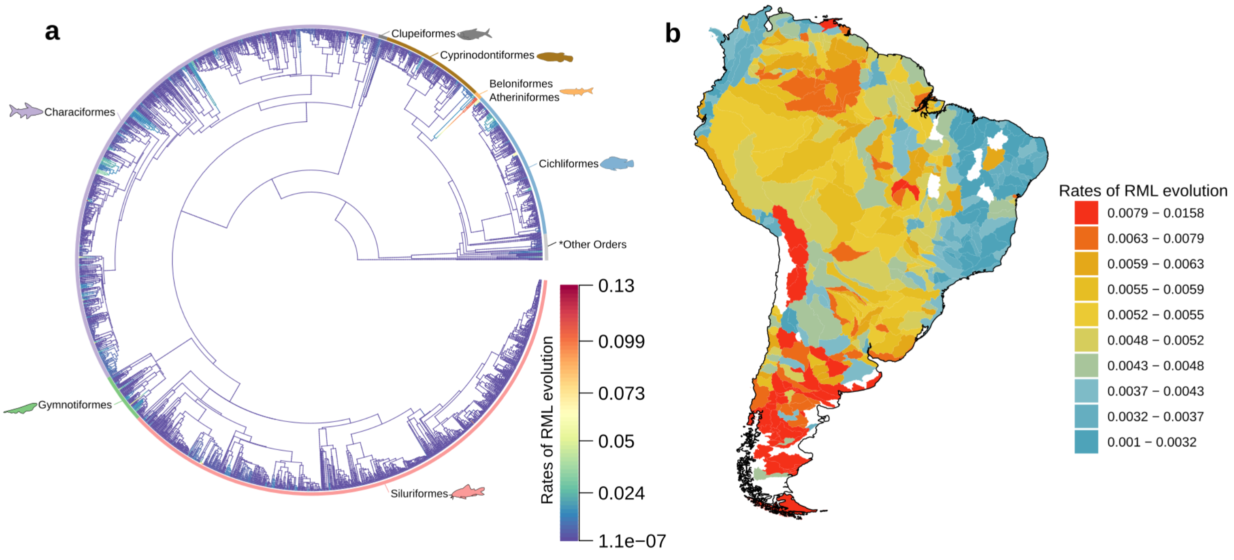

Figure S30. Temporal and geographic patterns of morphological evolution. Rates of relative maxillary length (RML) evolution measured across: **a** the entire phylogeny including 2,638 species, and **b** South American sub-basins. Evolutionary rates of RML were estimated using the BAMM 'trait' module. Evolutionary rates in sub-basins were calculated by averaging BAMM tip rates of co-occurring species.

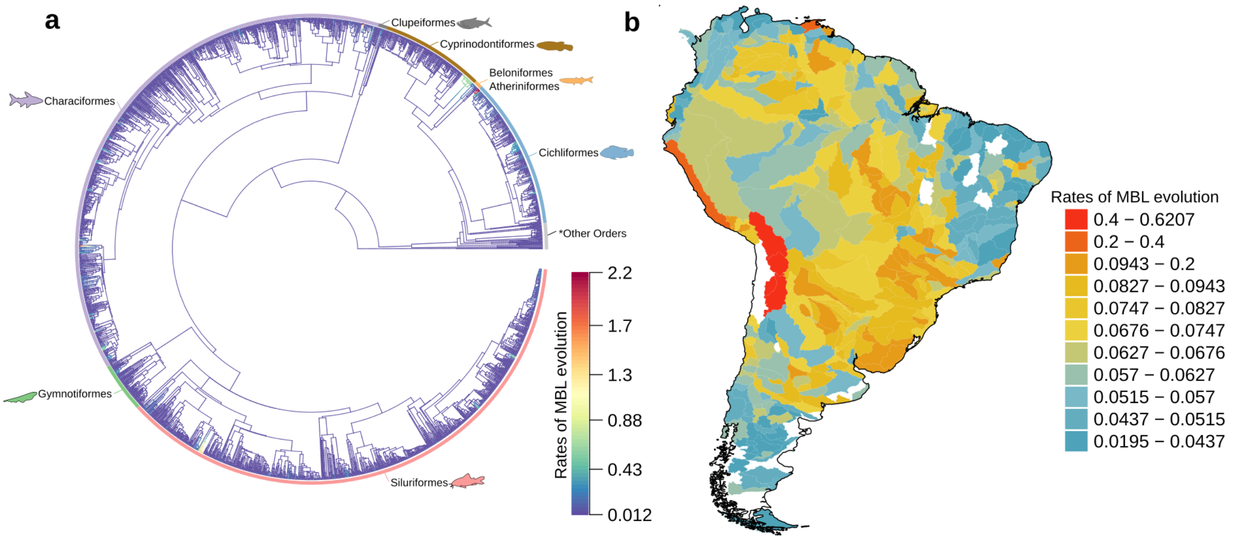

Figure S31. Temporal and geographic patterns of morphological evolution. Rates of maximum body length (MBL) evolution measured across: **a** the entire phylogeny including 2,638 species, and **b** South American sub-basins. Evolutionary rates of MBL were estimated using the BAMM ‘trait’ module. Evolutionary rates in sub-basins were calculated by averaging BAMM tip rates of co-occurring species.

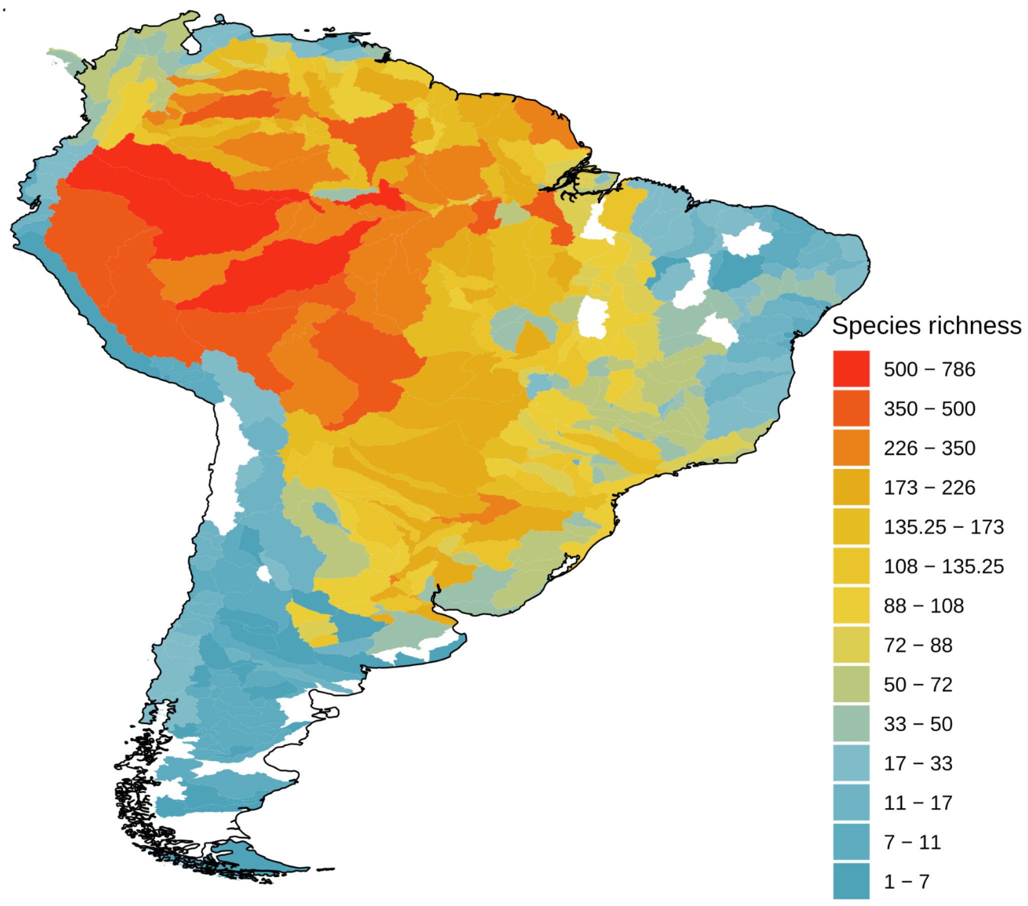

Figure S32. Species richness across sub-drainage basins in South America. Species richness was calculated as the total number of fish species co-occurring in each sub-basin. Red indicates the highest species richness while blue indicates the lowest.

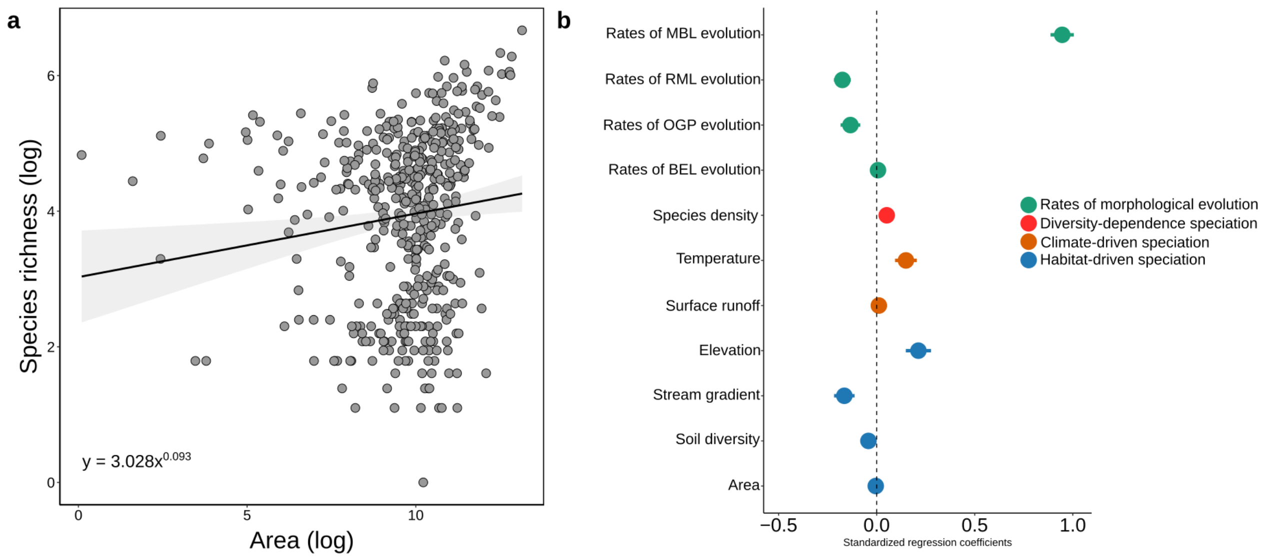

Figure S33. Reassessment of the importance of species diversity and area in the context of species-area relationship. Species density (SD) was calculated using the power function: SD = SR/Az, where SR is the number of species in area A, and z is the species-area scaling exponent (i.e., slope of the species-area regression). **a** The regression between species richness and sub-basin area resulted in a scaling exponent of 0.093. Both species richness and area were log-transformed. **b** Multiple linear regression showing standardized coefficients and their 95% confidence interval for each predictor. Note that the effects of species diversity (measured as species density) and area remained qualitatively similar to those in the main text (see Figure 2). Abbreviations for rates of morphological evolution: body elongation (BEL), maximum body length (MBL), oral gape position (OGP), and relative maxillary length (RML).

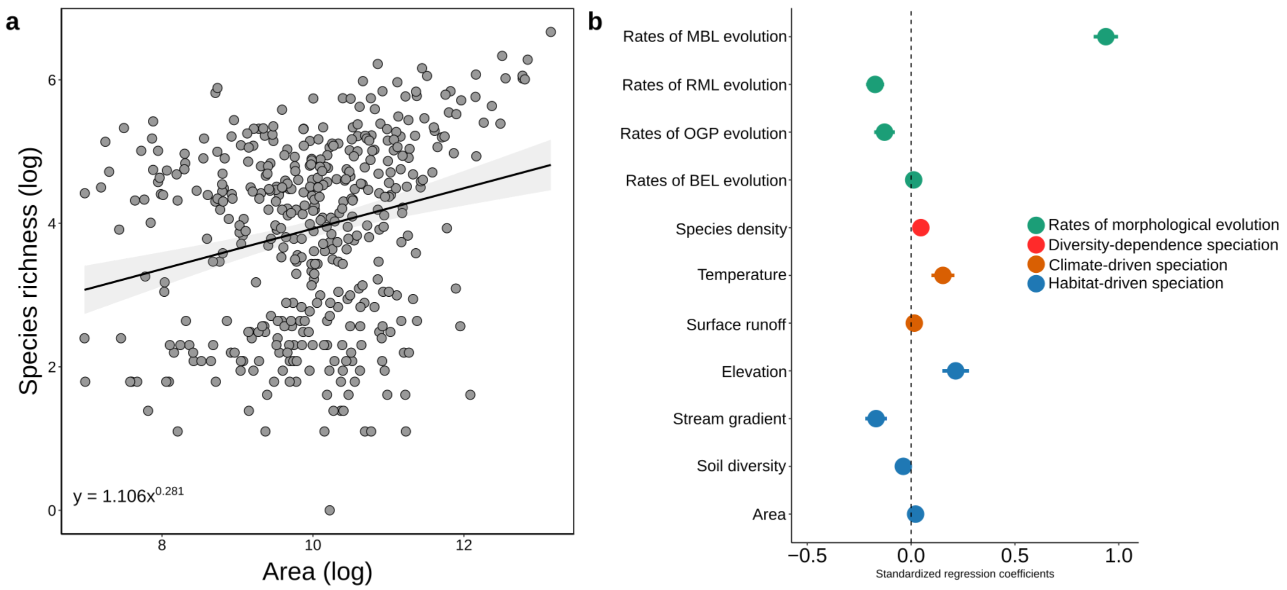

Figure S34. Reassessment of the importance of species diversity and area in the context of species-area relationship. Species density (SD) was calculated using the power function: SD = SR/Az, where SR is the number of species in area A, and z is the species-area scaling exponent (i.e., slope of the species-area regression). **a** Regression between species richness and sub-basin area after removing sub-basins with an area <100 km². This yielded a scaling exponent of 0.281, and both species richness and area were log-transformed. **b** Multiple linear regression showing standardized coefficients and their 95% confidence interval for each predictor. Note that the effects of species diversity (measured as species density) and area remained qualitatively similar to those in the main text (see Figure 2). Abbreviations for rates of morphological evolution: body elongation (BEL), maximum body length (MBL), oral gape position (OGP), and relative maxillary length (RML).

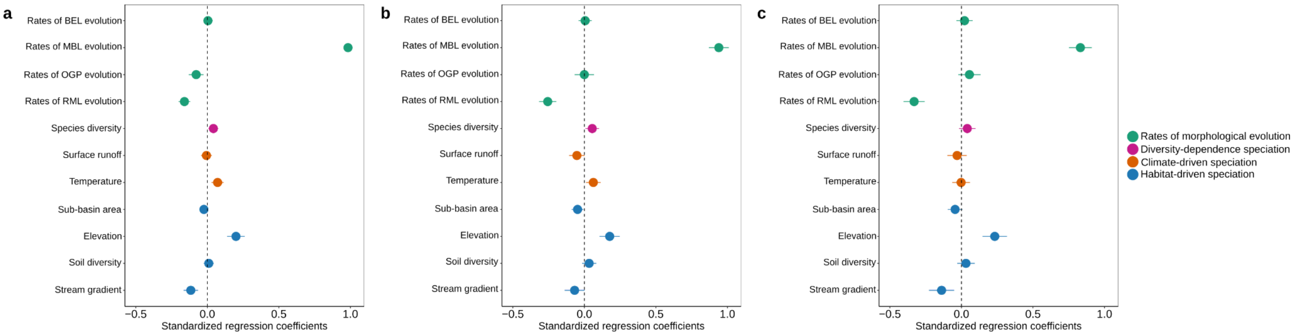

Figure S35. Relative importance of multiple predictors after excluding sub-basins with low species richness. Standardized regression coefficients and their 95% confidence intervals are presented for: **a** sub-basins with more than 10 species, **b** sub-basins with more than 15 species, and **c** sub-basins with more than 20 species. Abbreviations for rates of morphological evolution: body elongation (BEL), maximum body length (MBL), oral gape position (OGP), and relative maxillary length (RML).

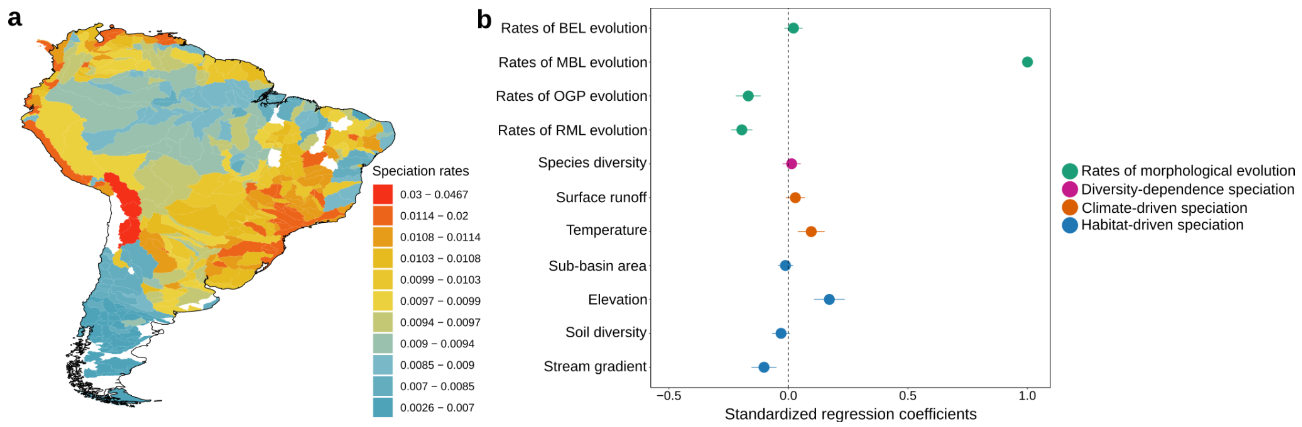

Figure S36. Spatial patterns of speciation rates weighted by species range size and the relative importance of multiple predictors. a Spatial distribution of speciation rates after being weighted by species range size. The congruence with the patterns in the main text (Figure 1b) is noticeable. **b** Standardized regression coefficients and their 95% confidence intervals of each predictor on speciation rates weighted by species range size. Abbreviations for rates of morphological evolution: body elongation (BEL), maximum body length (MBL), oral gape position (OGP), and relative maxillary length (RML).

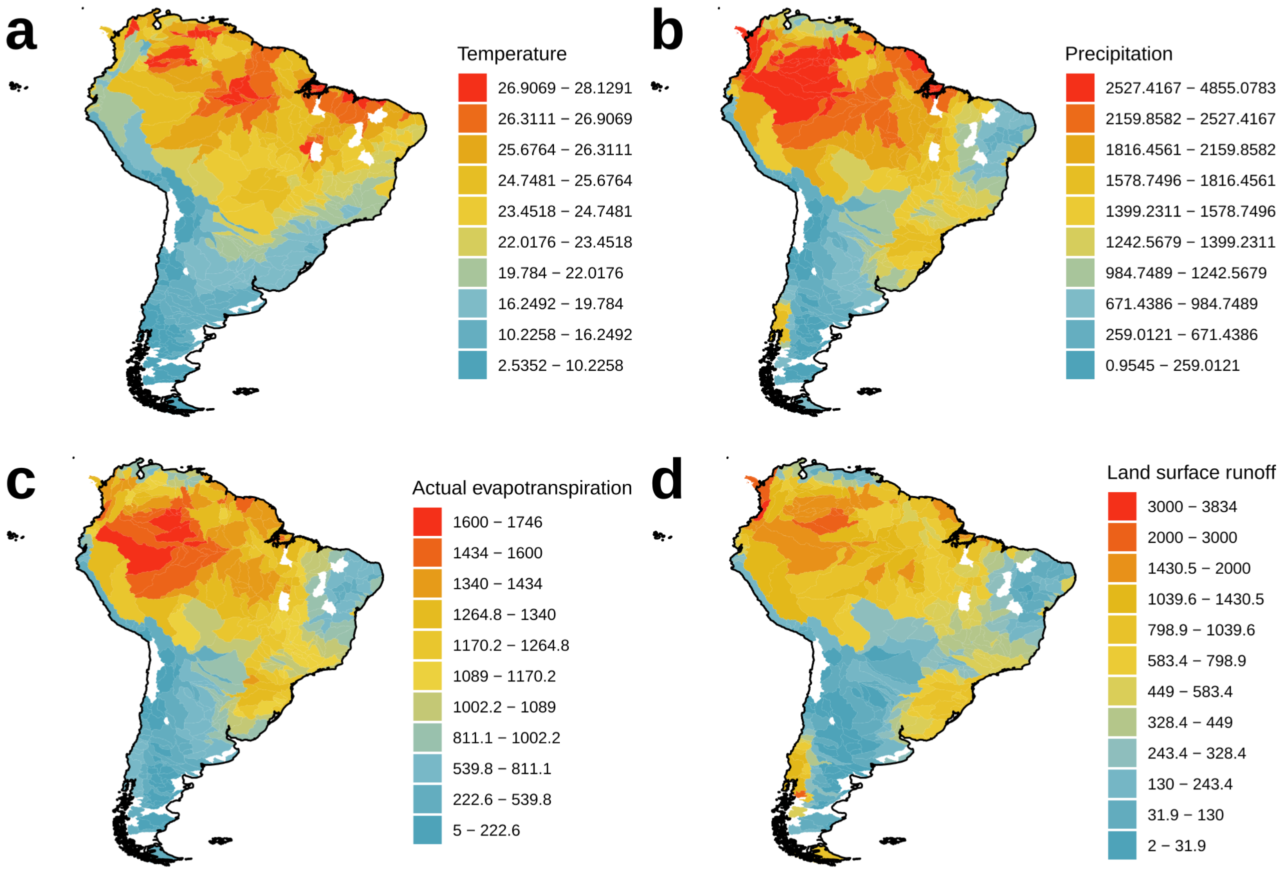

Figure S37. Variation in abiotic variables associated with the 'climate-driven' hypothesis across sub-basins. a Annual mean temperature. **b** Annual mean precipitation. **c** Actual evapotranspiration. **d** Land surface runoff. For further details, refer to the Methods section and Supplementary Table 3.

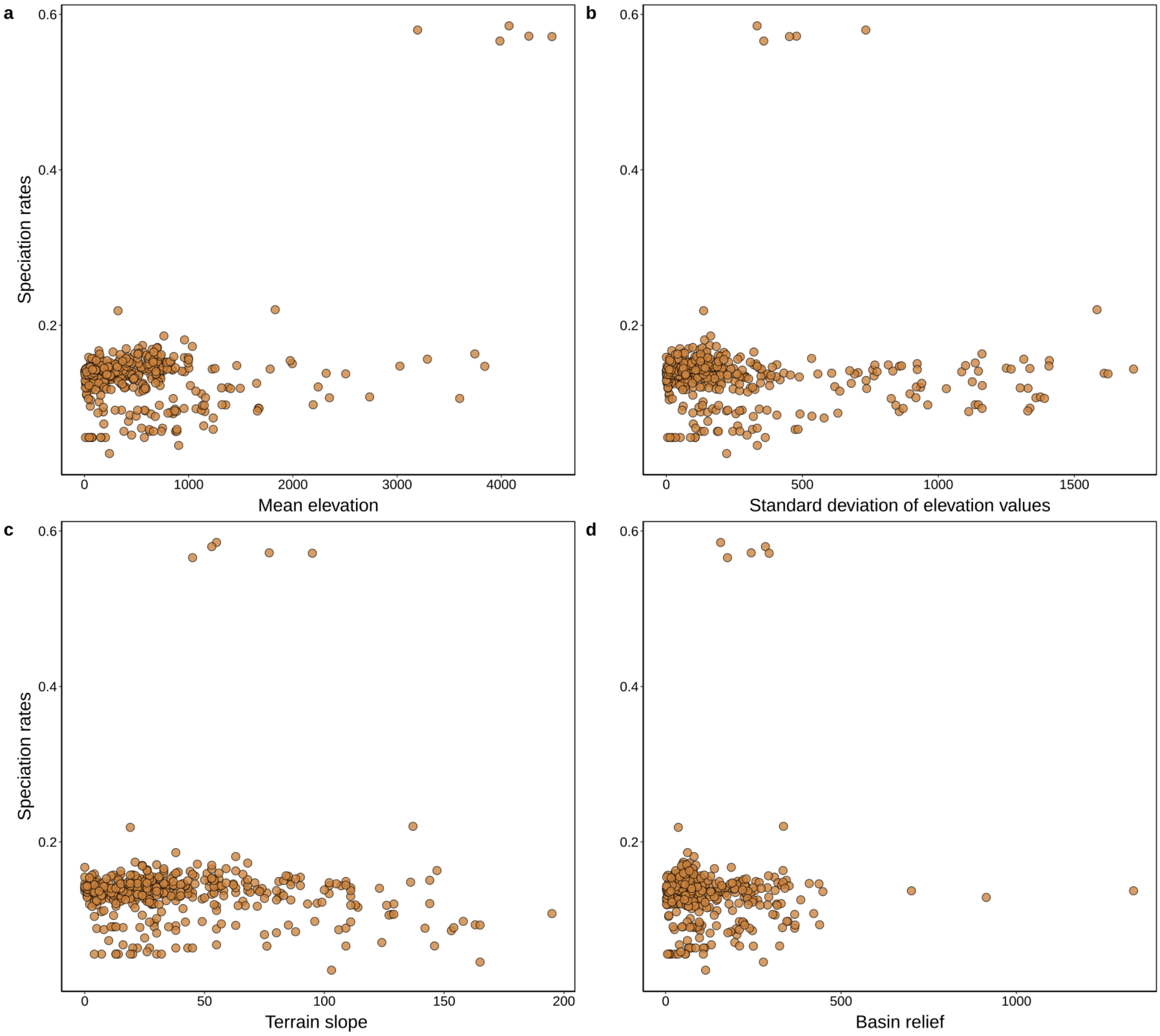

Figure S38. Importance of topographic complexity metrics on speciation rates. The relationship between speciation rates and alternative metrics of topographic complexity are shown: **a** mean elevation (Pearson's r = 0.472, $p < 0.001$), **b** standard deviation of elevation values (r = 0.011, p = 0.811), **c** terrain slope (r = -0.013, p = 0.773), and **d** basin relief (r = 0.042, p = 0.367). Each point represents an estimate obtained for a South American sub-basin. The standard deviation of elevation values was obtained from the WorldClim Version 1 database at a resolution of 2.5 arc-minutes (Hijmans et al., 2005). Terrain slope data were extracted from the HydroATLAS database at a resolution of 15 arc-seconds (Linke et al., 2019). Basin relief data were obtained from Shen et al. (2017).

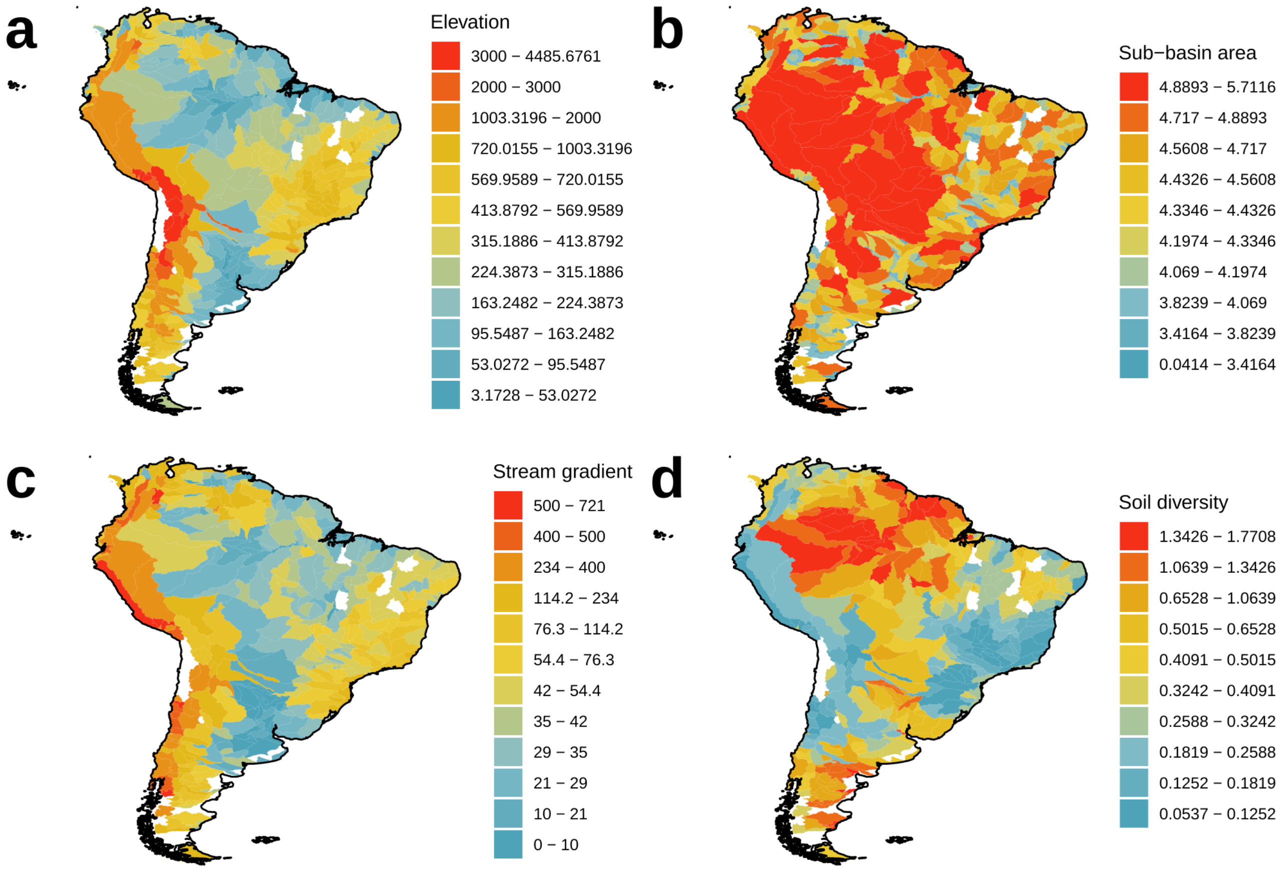

Figure S39. Variation in abiotic variables associated with the 'habitat-driven' hypothesis across sub-basins. The following variables are shown: **a** mean elevation, **b** geographic area, **c** stream gradient, and **d** soil diversity. See the Methods section and Supplementary Table 3 for more details.

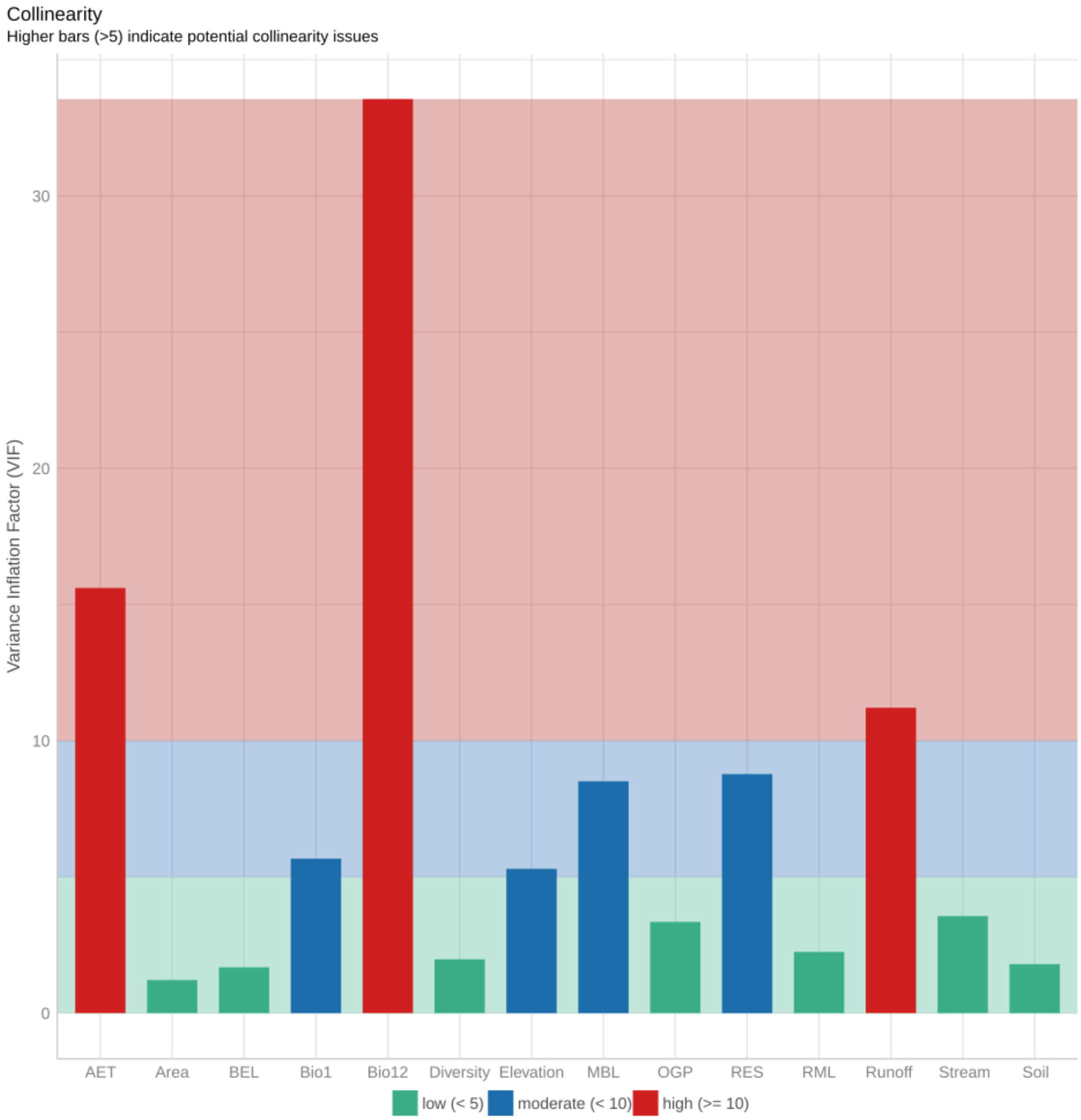

Figure S40. Verification of collinearity among predictors using the variance inflation factor (VIF). Three variables, namely RES (rates of relative eye size evolution), Bio12 (annual mean precipitation), and AET (actual evapotranspiration), showed high VIF values and were excluded from downstream analyses. The abbreviations used are as follows: AET (actual evapotranspiration), BEL (rates of body elongation evolution), Bio1 (annual mean temperature), Bio12 (annual mean precipitation), Diversity (species richness), MBL (rates of maximum body length evolution), OGP (rates of oral gape position evolution), RES (rates of relative eye size evolution), Runoff (land surface runoff), Stream (stream gradient), and Soil (soil diversity).

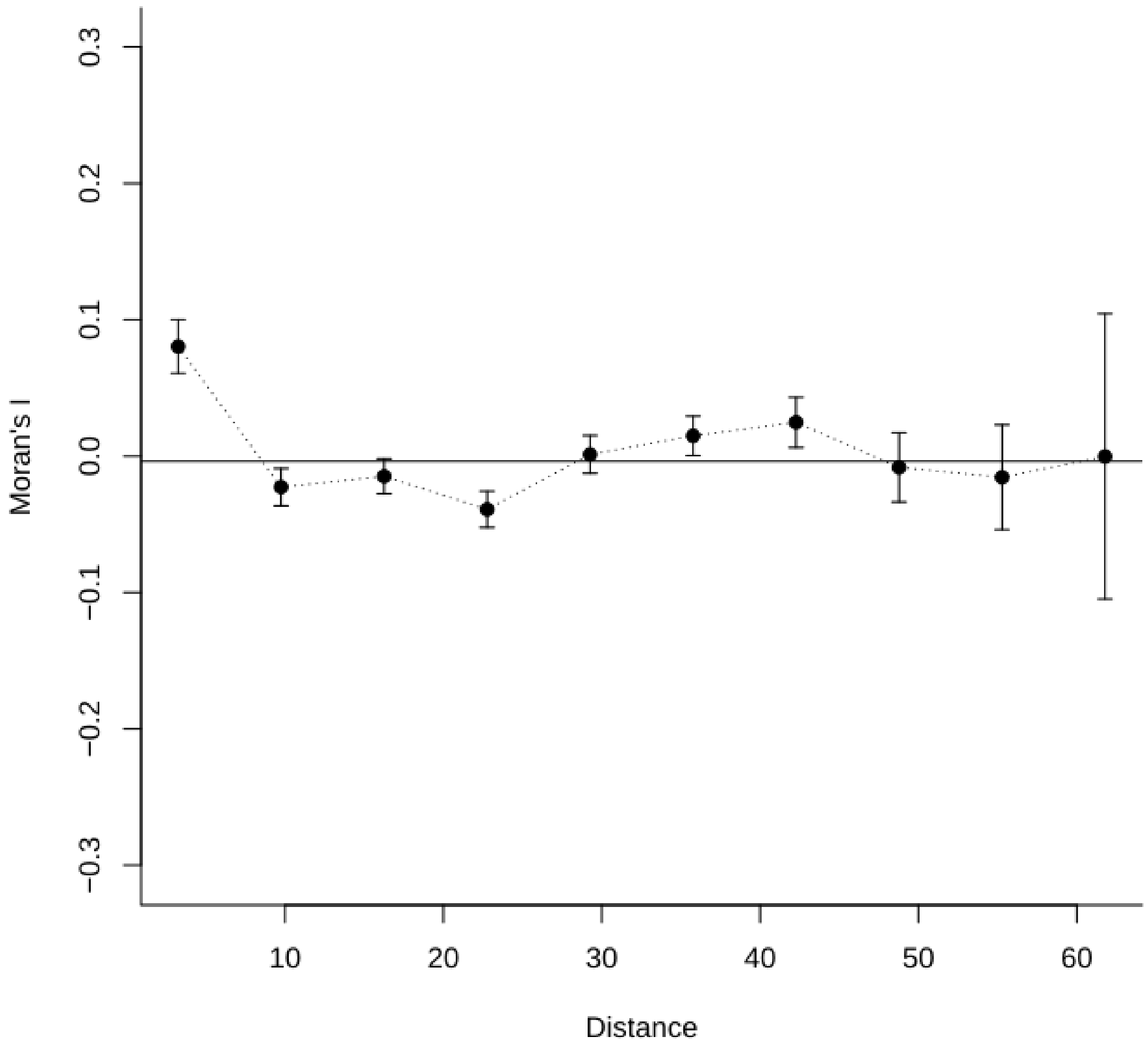

Figure S41. Correlogram of Moran's I values for the residuals of a multiple regression model that incorporates eleven biotic and abiotic predictors. Moran's I is a spatial autocorrelation statistic that measures the degree of similarity between the values of a variable at different locations. The correlogram depicts the correlation values of the residuals for different distance intervals, which indicate low spatial structure (Global *Moran's I* = -0.003, *P* = 0.423).

Supplementary Tables

Table S1. Results of multiple linear regression analysis evaluating the effects of eleven biotic and abiotic variables on speciation rates. Summary statistics include the Variance Inflation Factor (VIF), standardized regression coefficients (Std slope), t-value, and P-value (*P < 0.05, **P < 0.001). Predictors with statistically non-significant relationships with speciation rates are denoted by 'NS'.

Variable	VIF	Std slope	t-value	*P*-value
Rates of body elongation evolution	1.627	0.008	0.451	0.652^{NS}
Rates of maximum body length evolution	4.198	0.943	31.061	< 0.001**
Rates of oral gape position evolution	2.969	-0.130	-5.112	< 0.001**
Rates of relative maxillary length evolution	2.208	-0.176	-7.990	< 0.001**
Species diversity	1.785	0.046	2.338	0.002*
Surface runoff	1.906	0.013	0.659	0.510^{NS}
Temperature	3.656	0.150	5.319	< 0.001**
Area	1.193	-0.014	-0.893	0.372^{NS}
Elevation	4.893	0.213	6.514	< 0.001**
Soil diversity	1.664	-0.041	-2.157	0.031*
Stream gradient	3.246	-0.166	-6.245	< 0.001**

Table S2. The proportion of species sampled in the 20 most diverse genera.

Genus	Species number	Proportion sampled (%)
Corydoras	175	70.85
Trichomycterus	169	10.65
Hyphessobrycon	157	31.84
Hypostomus	153	39.86
Astyanax	124	25.80
Crenicichla	95	63.15
Apistogramma	94	53.19
Moenkhausia	92	45.65
Pimelodella	83	42.68
Leporinus	80	52.50
Creagrutus	75	66.66
Ancistrus	73	41.09
Characidium	70	45.71
Rineloricaria	66	28.78
Poecilia	57	15.78
Anablepsoides	55	56.36
Bryconamericus	54	31.48
Hypsolebias	52	28.84
Hemibrycon	51	29.41
Chaetostoma	48	33.33

Table S3. Biotic and abiotic factors used to explain the spatial variation in speciation rates. Abbreviations for rates of morphological evolution: body elongation (BEL), maximum body length (MBL), oral gape position (OGP), and relative maxillary length (RML).

Group	Variable	Transformation	Source	Reference
Biotic	Rates of BEL evolution<br>Rates of MBL evolution<br>Rates of OGP evolution<br>Rates of RMLevolution<br>Rates of RES evolution*	scale	Fishmorph	Brosse et al., 2021**
	Species diversity	scale	-	-
Abiotic: Climate-driven	Temperature	scale	WorldClim	Hijmans et al., 2005
	Precipitation*	-	WorldClim	Hijmans et al., 2005
	Actual evapotranspiration*	-	HydroATLAS	Linke et al., 2019
	Surface runoff	scale	HydroATLAS	Linke et al., 2019
Abiotic: Habitat-driven	Area	log10, scale	HydroATLAS	Linke et al., 2019
	Elevation	scale	WorldClim	Hijmans et al., 2005
	Soil diversity***	scale	HydroATLAS	Linke et al., 2019
	Stream gradient	scale	HydroATLAS	Linke et al., 2019

* Variables removed from the multiple regression due to the high multicollinearity

** Raw morphological data were obtained from Brosse et al., 2021 and later used to quantify the evolutionary dynamics using BAMM program

*** Soil variables used: clay fraction, silt fraction, sand fraction, organic carbon content, soil water content, lithological classes, karst area extent, soil erosion

Supplementary references

1. Louca, S. & Pennell, M. W. Extant timetrees are consistent with a myriad of diversification histories. *Nature* 1–4 (2020) doi:10.1038/s41586-020-2176-1.
2. Vasconcelos, T., O'Meara, B. C. & Beaulieu, J. M. A flexible method for estimating tip diversification rates across a range of speciation and extinction scenarios. *Evolution* **76**, 1420–1433 (2022).
3. Siqueira, A. C., Morais, R. A., Bellwood, D. R. & Cowman, P. F. Trophic innovations fuel reef fish diversification. *Nat. Commun.* **11**, 2669 (2020).
4. Rabosky, D. L. Automatic Detection of Key Innovations, Rate Shifts, and Diversity-Dependence on Phylogenetic Trees. *PLOS ONE* **9**, e89543 (2014).
5. Title, P. O. & Rabosky, D. L. Tip rates, phylogenies and diversification: What are we estimating, and how good are the estimates? *Methods Ecol. Evol.* **10**, 821–834 (2019).
6. Cooney, C. R. & Thomas, G. H. Heterogeneous relationships between rates of speciation and body size evolution across vertebrate clades. *Nat. Ecol. Evol.* (2020) doi:10.1038/s41559-020-01321-y.
7. Cassemiro, F. A. S. *et al.* Landscape dynamics and diversification of the megadiverse South American freshwater fish fauna. *Proc. Natl. Acad. Sci.* **120**, e2211974120 (2023).
8. Rabosky, D. L. *et al.* An inverse latitudinal gradient in speciation rate for marine fishes. *Nature* **559**, 392 (2018).
9. Reznick, D. N., Furness, A. I., Meredith, R. W. & Springer, M. S. The origin and biogeographic diversification of fishes in the family Poeciliidae. *PLOS ONE* **12**, e0172546 (2017).
10. Burress, E. D. & Tan, M. Ecological opportunity alters the timing and shape of adaptive radiation. *Evolution* **71**, 2650–2660 (2017).
11. Melo, B. F. *et al.* Accelerated Diversification Explains the Exceptional Species

Richness of Tropical Characoid Fishes. *Syst. Biol.* **71**, 78–92 (2022).

12. Craig, J. M., Kumar, S. & Hedges, S. B. Limitations of Phylogenomic Data Can Drive Inferred Speciation Rate Shifts. *Mol. Biol. Evol.* **39**, msac038 (2022).
13. Rabosky, D. L. Phylogenies and Diversification Rates: Variance Cannot Be Ignored. *Syst. Biol.* **68**, 538–550 (2019).
14. Rabosky, D. L. *et al.* Rates of speciation and morphological evolution are correlated across the largest vertebrate radiation. *Nat. Commun.* **4**, 1958 (2013).
15. Froese, R. & Pauly, D. FishBase: World Wide Web electronic publication. www.fishbase.org (2022).
16. Dengler, J. Which function describes the species–area relationship best? A review and empirical evaluation. *J. Biogeogr.* **36**, 728–744 (2009).
17. Harvey, M. G. *et al.* The evolution of a tropical biodiversity hotspot. *Science* **370**, 1343–1348 (2020).
18. Karger, D. N. *et al.* Climatologies at high resolution for the earth's land surface areas. *Sci. Data* **4**, 170122 (2017).
19. Morlon, H. *et al.* RPANDA: an R package for macroevolutionary analyses on phylogenetic trees. *Methods Ecol. Evol.* **7**, 589–597 (2016).
20. Zachos, J. C., Dickens, G. R. & Zeebe, R. E. An early Cenozoic perspective on greenhouse warming and carbon-cycle dynamics. *Nature* **451**, 279–283 (2008).
21. Linke, S. *et al.* Global hydro-environmental sub-basin and river reach characteristics at high spatial resolution. *Sci. Data* **6**, 283 (2019).
22. Diniz-Filho, J. A. F., Bini, L. M. & Hawkins, B. A. Spatial autocorrelation and red herrings in geographical ecology. *Glob. Ecol. Biogeogr.* **12**, 53–64 (2003).
23. Legendre, P. Spatial Autocorrelation: Trouble or New Paradigm? *Ecology* **74**, 1659–1673 (1993).
24. Dehon, C., Gassner, M. & Verardi, V. Beware of 'Good' Outliers and Overoptimistic

Conclusions*. *Oxf. Bull. Econ. Stat.* **71**, 437–452 (2009).

25. Di Genova, A. *et al.* Genome sequencing and transcriptomic analysis of the Andean killifish Orestias ascotanensis reveals adaptation to high-altitude aquatic life. *Genomics* **114**, 305–315 (2022).
26. Guerrero-Jiménez, C. J. *et al.* Pattern of genetic differentiation of an incipient speciation process: The case of the high Andean killifish Orestias. *PLOS ONE* **12**, e0170380 (2017).
27. Lehner, B. & Grill, G. Global river hydrography and network routing: baseline data and new approaches to study the world's large river systems. *Hydrol. Process.* **27**, 2171–2186 (2013).
28. Jetz, W., Thomas, G. H., Joy, J. B., Hartmann, K. & Mooers, A. O. The global diversity of birds in space and time. *Nature* **491**, 444–448 (2012).
29. Reis, R. E. *et al.* Fish biodiversity and conservation in South America. *J. Fish Biol.* **89**, 12–47 (2016).
30. Rabosky, D. L. *et al.* BAMMtools: an R package for the analysis of evolutionary dynamics on phylogenetic trees. *Methods Ecol. Evol.* **5**, 701–707 (2014).
31. Plummer, M., Best, N., Cowles, K. & Vines, K. CODA: convergence diagnosis and output analysis for MCMC. *R News* **6**, 7–11 (2006).
32. Kembel, S. W. *et al.* Picante: R tools for integrating phylogenies and ecology. *Bioinformatics* **26**, 1463–1464 (2010).
33. Beaulieu, J. M. & O'Meara, B. C. Detecting Hidden Diversification Shifts in Models of Trait-Dependent Speciation and Extinction. *Syst. Biol.* **65**, 583–601 (2016).
34. Rabosky, D. L. Extinction Rates Should Not Be Estimated from Molecular Phylogenies. *Evolution* **64**, 1816–1824 (2010).
35. Mitchell, J. S., Etienne, R. S. & Rabosky, D. L. Inferring Diversification Rate Variation From Phylogenies With Fossils. *Syst. Biol.* **68**, 1–18 (2019).

36. Bivand, R., Keitt, T., Rowlingson, B., Pebesma, E., Sumner, M., Hijmans, R., ... & Bivand, M. R. Package ‘rgdal’. Bindings for the Geospatial Data Abstraction Library. (2015).
37. Debastiani, V. J. & Pillar, V. D. SYNCSA—R tool for analysis of metacommunities based on functional traits and phylogeny of the community components. *Bioinformatics* **28**, 2067–2068 (2012).
38. Brosse, S. *et al.* FISHMORPH: A global database on morphological traits of freshwater fishes. *Glob. Ecol. Biogeogr.* **n/a**,.
39. Claverie, T. & Wainwright, P. C. A Morphospace for Reef Fishes: Elongation Is the Dominant Axis of Body Shape Evolution. *PLOS ONE* **9**, e112732 (2014).
40. Toussaint, A. *et al.* Non-native species led to marked shifts in functional diversity of the world freshwater fish faunas. *Ecol. Lett.* **21**, 1649–1659 (2018).
41. Corral-López, A., Garate-Olaizola, M., Buechel, S. D., Kolm, N. & Kotrschal, A. On the role of body size, brain size, and eye size in visual acuity. *Behav. Ecol. Sociobiol.* **71**, 179 (2017).
42. Kopf, R. K., Yen, J. D. L., Nimmo, D. G., Brosse, S. & Villéger, S. Global patterns and predictors of trophic position, body size and jaw size in fishes. *Glob. Ecol. Biogeogr.* **30**, 414–428 (2021).
43. Knouft, J. H. & Page, L. M. The Evolution of Body Size in Extant Groups of North American Freshwater Fishes: Speciation, Size Distributions, and Cope’s Rule. *Am. Nat.* **161**, 413–421 (2003).
44. Benson, R. B. J., Godoy, P., Bronzati, M., Butler, R. J. & Gearty, W. Reconstructed evolutionary patterns for crocodile-line archosaurs demonstrate impact of failure to log-transform body size data. *Commun. Biol.* **5**, 1–4 (2022).
45. Goolsby, E. W., Bruggeman, J. & Ané, C. Rphylopars: fast multivariate phylogenetic comparative methods for missing data and within-species variation. *Methods Ecol.*

Evol. **8**, 22–27 (2017).

46. Penone, C. *et al.* Imputation of missing data in life-history trait datasets: which approach performs the best? *Methods Ecol. Evol.* **5**, 961–970 (2014).
47. Venditti, C., Meade, A. & Pagel, M. Multiple routes to mammalian diversity. *Nature* **479**, 393–396 (2011).
48. Baker, J., Meade, A., Pagel, M. & Venditti, C. Positive phenotypic selection inferred from phylogenies. *Biol. J. Linn. Soc.* **118**, 95–115 (2016).
49. Allen, A. P., Gillooly, J. F., Savage, V. M. & Brown, J. H. Kinetic effects of temperature on rates of genetic divergence and speciation. *Proc. Natl. Acad. Sci.* **103**, 9130–9135 (2006).
50. Storch, D., Bohdalková, E. & Okie, J. The more-individuals hypothesis revisited: the role of community abundance in species richness regulation and the productivity-diversity relationship. *Ecol. Lett.* **21**, 920–937 (2018).
51. Hijmans, R. J. & Van Etten, J., Cheng, J., Mattiuzzi, M., Sumner, M., Greenberg, J. A., ... & Hijmans, M. R. J. The raster package. (2015).
52. Leprieur, F. *et al.* Partitioning global patterns of freshwater fish beta diversity reveals contrasting signatures of past climate changes. *Ecol. Lett.* **14**, 325–334 (2011).
53. Tedesco, P. A. *et al.* Patterns and processes of global riverine fish endemism. *Glob. Ecol. Biogeogr.* **21**, 977–987 (2012).
54. Oberdorff, T. *et al.* Unexpected fish diversity gradients in the Amazon basin. *Sci. Adv.* **5**, eaav8681.
55. Dias, M. S. *et al.* Global imprint of historical connectivity on freshwater fish biodiversity. *Ecol. Lett.* **17**, 1130–1140 (2014).
56. Carvajal-Quintero, J. *et al.* Drainage network position and historical connectivity explain global patterns in freshwater fishes' range size. *Proc. Natl. Acad. Sci.* **116**, 13434–13439 (2019).

57. Dixon, P. VEGAN, a package of R functions for community ecology. *J. Veg. Sci.* **14**, 927–930 (2003).

58. Fox, J., Weisberg, S., Adler, D. & Monette, G. Package ‘car’. (2012).

59. Groemping, U. Relative Importance for Linear Regression in R: The Package relaimpo. *J. Stat. Softw.* **17**, 1–27 (2007).

60. Grömping, U. Variable importance in regression models. *WIREs Comput. Stat.* **7**, 137–152 (2015).
